# Tubulin flux at spastin-induced nanodamage sites regulates microtubule rescue frequency and EB1 lifetimes

**DOI:** 10.1073/pnas.2517683123

**Published:** 2026-05-26

**Authors:** Ewa Szczesna, Jeffrey O. Spector, Stephanie L. Sarbanes, Jiayi Chen, Agnieszka Szyk, Antonina Roll-Mecak

**Affiliations:** ^a^Cell Biology and Biophysics Unit, National Institute of Neurological Disorders and Stroke, Bethesda, MD 20892; ^b^Biochemistry and Biophysics Center, National Heart, Lung and Blood Institute, Bethesda, MD 20892

**Keywords:** microtubules, severing enzyme, microtubule dynamics, microtubule repair, GTP–tubulin islands

## Abstract

Severing enzymes nanodamage microtubules by extracting tubulin subunits. This extraction is accompanied by spontaneous repair with soluble tubulin. Here, we show that GTP-, and not GDP–tubulin, incorporates preferentially at nanodamage sites where it recruits end binding protein-1 (EB1). Using GTP hydrolysis-defective recombinant tubulin mutants we show that the tubulin GTPase is a timer for EB1 association at repair sites. Tubulin on-rate controls both the size and lifetime of the spastin-catalyzed GTP-islands which promote rescues. Consistent with this, first, rescue rates in the presence of repair vary steeply with tubulin concentration, unlike in the absence of repair, and second, spastin-catalyzed lattice remodeling, and not passive binding of the enzyme alone, increases rescue rates. Furthermore, we show that, when overexpressed at low levels in cells, spastin catalyzes microtubule repair and affects microtubule growth. Thus, microtubule severing proceeds through GTP–tubulin repair intermediates both in vitro and in cells. Our work demonstrates that severing enzymes exert their effects on microtubule function through tubulin extraction and repair and strongly supports microtubule dynamics regulation through tubulin dimer exchange along the lattice.

## Significance

Microtubule severing enzymes are critical in diverse cellular processes. Reconstitution experiments revealed that severing enzymes extract tubulin subunits and catalyze repair with fresh tubulin, creating islands formed of tubulin bound to guanosine triphosphate (GTP) along microtubules that regulate their dynamics. Here, we show that the rate of GTP hydrolysis on tubulin controls the binding of effectors at repair sites and that tubulin flux at repair sites regulates the frequency of rescues, the transitions from microtubule depolymerization to regrowth. Furthermore, we show that severing enzymes catalyze microtubule repair in cells with effects on microtubule growth. Our results add to the growing body of evidence that shows that microtubule dynamics are controlled not only by addition of tubulin at the tips but also along the microtubule shaft.

Microtubules are cylindrical noncovalent polymers of αβ-tubulin heterodimers. They are essential for cell division, motility, intracellular transport, and signaling. An array of cellular factors orchestrates the formation of microtubule networks with distinct dynamics and morphologies. Microtubule severing enzymes spastin and katanin are AAA ATPases that cut microtubules along their lengths in an ATP hydrolysis–dependent manner (1). They function in cilia biogenesis, cell division, neurogenesis, and phototropism, and their mutation is associated with nervous system disorders (1, 2). Spastin is mutated in hereditary spastic paraplegia, a neurodegenerative disease characterized by “dying back” of the long axons of cortical motor neurons (3), and spastin disease mutants have impaired microtubule severing (4, 5). Katanin mutations cause microcephaly (6, 7).

Microtubules grow through the addition of GTP–tubulin at their ends. GTP converts to GDP as tubulin integrates into the lattice. Thus, the microtubule is composed mostly of GDP–tubulin, but because the GTPase rate is slower than the rate of GTP–tubulin addition (8–12), its tip is enriched in GTP–tubulin. The GDP–microtubule lattice is unstable and depolymerizes rapidly if exposed, while the GTP-lattice is stable. Its presence at the microtubule tip as a “GTP-cap” stabilizes the microtubule against depolymerization. Erosion of the GTP cap triggers catastrophe, a switch from growth to depolymerization (13–15). Both spastin and katanin sever microtubules by extracting tubulin subunits out of the microtubule in an ATPase-dependent fashion. This extraction creates nanodamage sites along the microtubule that can eventually, but not always, become severing sites visible at the mesoscale (16). While not visible at the resolution of a light microscope, these nanodamage sites were visualized directly by electron microscopy (16). The extraction of tubulin is accompanied by spontaneous repair of the nanodamage with soluble tubulin (16). Thus, the repair that accompanies tubulin extraction by severing enzymes creates a “GTP-island” in a microtubule otherwise composed overwhelmingly of GDP–tubulin (16). This lattice repair is also associated with an increase in rescue events (the switch from microtubule depolymerization to regrowth) and an increase in the stability of newly severed plus ends, which in the absence of the repair catalyzed by the enzyme, depolymerize spontaneously (16). Thus, severing generates new microtubule plus ends that are more stable and can support rapid regrowth (16). Such a mechanism of microtubule-based microtubule amplification was proposed earlier (17, 18) and provides a mechanistic framework for interpreting in vivo phenotypes associated with spastin and katanin dysfunction which lead to a reduction in microtubule density in several model systems (19), e.g., spastin mutant flies have fewer microtubules at the neuromuscular junction (20) and katanin loss leads to a reduction in microtubule numbers in *Arabidopsis* cortical arrays (21).

Several fundamental questions remain unanswered: i) Does the nucleotide state of tubulin influence incorporation at repair sites? GDP–tubulin can incorporate slowly at microtubule ends in vitro (22), however a repair site has a different morphology than a growing microtubule end, and GTP–tubulin, unlike at the microtubule end, needs to incorporate into a preexisting compacted GDP lattice with a different spacing than the expanded GTP-lattice (23, 24); ii) Is GTP hydrolysis required for tubulin incorporation at the repair site?; iii) the repair site is recognized by End binding protein (EB1) (16). What features of the repair site does EB1 recognize? Does EB1 recognize the nucleotide state of the lattice, or does it recognize also interfaces exposed at nanodamage sites by the extraction process as proposed (25)? iv) What is the contribution of passive binding of the severing enzyme to the increase in rescue rates? Using laser ablation of GMPCPP capped microtubules in the presence of spastin and ATP or the nonhydrolyzable analog ATPγS we showed that a robust increase in rescue frequency requires active remodeling of the microtubule lattice (16). Subsequent work advanced an alternative model (26) in which rescues induced by spastin are due only to the passive binding of the enzyme to the microtubule (without tubulin extraction) and not repair; v) Finally, is the repair catalyzed by severing enzymes also operational in the more complex cellular environment where microtubules and tubulin are associated with many other proteins that could affect their extraction and incorporation into the lattice?

We now answer all these questions. Combining in vitro reconstitution with total internal reflection fluorescence (TIRF) and interference reflection (IR) microscopy, we show that GTP- and not GDP–tubulin preferentially incorporates at nanodamage sites, and that the effect on rescues is dependent on the flux of GTP–tubulin at the repair site, with maximal rescue rates achieved through microtubule lattice healing and not passive enzyme binding. Using recombinant tubulin mutants defective in GTP hydrolysis we show that tubulin incorporation at damage sites does not require GTP hydrolysis and that EB1 lattice binding is facilitated by recognition of the GTP-state of the incorporated tubulin and not ultrastructural features of the damaged lattice. However, GTP hydrolysis rate is the timer for EB1 association with repair sites. Lastly, we show that spastin overexpression induces microtubule repair and the redistribution of EB1 along microtubules in cells. Thus, the tubulin extraction and repair mechanism with GTP–tubulin we described for spastin in vitro is also operational in the complex cellular environment. This has implications for the recruitment at sites of spastin action of cellular factors that are sensitive to the nucleotide state of tubulin.

## Results

### GTP–Tubulin Incorporates Preferentially over GDP–Tubulin at Nanodamage Sites.

**Fig. 1.**
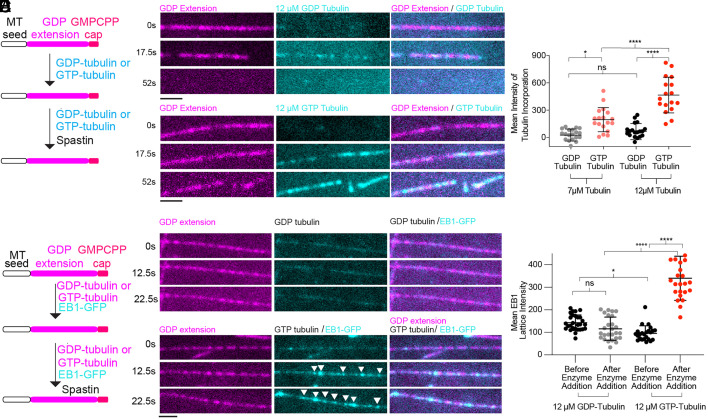
GTP–tubulin incorporates preferentially over GDP–tubulin at nanodamage sites. and is recognized by EB1. (*A*) Schematic of experiments in *B* and *C*. GDP microtubule extensions (magenta) were grown from GMPCPP seeds (black outline) and capped with GMPCPP tubulin (red). GDP– or GTP–tubulin (cyan) were perfused followed by spastin with GDP– or GTP–tubulin (*Materials and Methods*). (*B*) Time series of GMPCPP-capped GDP–microtubule extension (magenta) in the presence of 10 nM spastin and 12 µM GDP– (*Top*) or GTP–tubulin (*Bottom*) (cyan); (Scale bar, 2 µm.) See Movies S1 and S2. For panels with 7 µM GDP– or GTP–tubulin, see *SI Appendix*, Fig. S1*B* and Movies S3 and S4. (*C*) Background-corrected intensity of tubulin incorporated into spastin-mediated nanodamage sites along the microtubule in the presence of 7 µM GDP– or GTP–tubulin and 12 µM GDP– or GTP–tubulin 16 s after tubulin/spastin perfusion; n = 20, 18, 20, and 17 microtubules for 7 µM GDP–tubulin, 7 µM GTP–tubulin, 12 µM GDP–tubulin, or 12 µM GTP–tubulin, respectively, from 3 and 2 independent chambers, respectively. ****P < 0.0001, *P < 0.05, ns *P* > 0.05 by two-way ANOVA. (*D*) Schematic of experiments in *E* and *F*. GDP microtubule extensions (magenta) were polymerized from GMPCPP seeds (black outline) and capped with GMPCPP tubulin (red). GDP– or GTP–tubulin with EB1 (cyan) were perfused into the chamber followed by spastin with GDP– or GTP–tubulin and EB1 (*Materials and Methods*). (*E*) Images of GDP microtubule extensions (magenta) in the presence of 12 µM GDP– or GTP–tubulin, EB1–GFP (cyan), and 5 nM spastin at different times post perfusion. White arrows indicate EB1-GFP puncta. (Scale bar, 2 µm.) (*F*) Mean EB1 intensity on microtubules repaired with GDP– and GTP–tubulin; n = 28, 26, 24 and 24 microtubules for the GDP– (before and after enzyme addition) and GTP–tubulin (before and after enzyme addition) condition, respectively, from 3 and 2 independent experiments; ns, nonsignificant, ****P < 0.0001, *P < 0.05 by two-way ANOVA. Error bars, SD See Movies S5 and S6.

During polymerization, GTP–tubulin incorporates at microtubule ends. The lattice acts as an allosteric effector (27) and promotes the hydrolysis of GTP to GDP on β-tubulin, leading to lattice compaction and a change in twist (24, 28–30). Repair requires the integration of the tubulin subunit into a preexisting microtubule lattice. Therefore, the incorporated tubulin can have more lattice contacts initially (both lateral, between neighboring protofilaments, and longitudinal, within the same protofilament) than the tubulin incorporated into separated protofilaments at microtubule ends and where it initially makes only longitudinal contacts. However, because GTP and GDP lattices have different spacings and twists (24, 28, 29), repair creates a discontinuity in lattice parameters as GTP–tubulin is incorporated into the GDP–microtubule lattice unlike at microtubule ends where the incoming GTP–tubulin dimer is added to a GTP-bound tubulin subunit. Since all microtubule repair assays performed to date were performed with GTP–tubulin, we investigated the effect of the bound nucleotide, GTP or GDP, on repair (Fig. 1 *A*–*C*). We grew GDP–microtubules from a preexisting seed and capped them with GMPCPP-tubulin to suppress end dynamics (31). These capped microtubules were then exposed to 10 nM purified spastin (*SI Appendix*, Fig. S1*A*) and either 7 or 12 µM fluorescently labeled GTP- or GDP–tubulin. Spastin concentration was chosen such that the microtubules in the GDP–tubulin chambers could persist long enough after perfusion to allow refocusing and image acquisition. We observed GTP–tubulin incorporate all along the microtubule (Fig. 1*B*, *SI Appendix*, Fig. S1*B*–*D*, and Movies S1–S4). In contrast, we observed little incorporation of GDP–tubulin. At 7 µM GDP–tubulin, the signal for the incorporated GDP–tubulin is close to background (Fig. 1*C* and *SI Appendix*, Fig. S1 *B* and *C*). At 12 µM, incorporation of GDP–tubulin along the microtubule is visible above background (Fig. 1*B*) but ~4.5-fold lower than that for GTP–tubulin (Fig. 1*C*). GDP–tubulin addition at the ends of the GMPCPP caps is not visible in these conditions (Movie S1). Therefore, the additional lateral lattice contacts available at repair sites favor incorporation along the microtubule compared to ends. Robust GTP–tubulin incorporation is visible all along the lattice both at 7 and 12 µM tubulin (Fig. 1*B*, *SI Appendix*, Fig. S1 *C* and *D*, and Movies S2 and S4) and is higher at 12 µM tubulin (Fig. 1*C*), consistent with faster on-rate at repair sites at the higher tubulin concentration.

### EB1 Recognizes GTP–Tubulin Islands and Not the Damaged Lattice.

EBs recognize the GTP or GDP–Pi microtubule lattice (13, 25, 32–34) and track with the growing end which is enriched in these nucleotide states. While studies showed that EBs recognize GTP–tubulin at growing ends, including using tubulin GTP-hydrolysis defective mutants (13), it has been proposed that EBs also recognize ultrastructural features of the microtubule lattice, independent of the nucleotide state, leading to accumulation at microtubule damage sites which have exposed protofilaments (25). To discern between these two mechanisms of EB1 recruitment at repair sites in the context of microtubule severing, we imaged EB1-GFP on microtubules nanodamaged with spastin, in the presence of either GTP– or GDP–tubulin (Fig. 1*D*). We saw that EB1-GFP lattice binding does not increase on nanodamaged microtubules compared to undamaged ones (Fig. 1 *E* and *F* and Movie S5). However, mean EB1 lattice intensity increases ~threefold in the presence of soluble GTP–tubulin (Fig. 1*F*) with EB1 clearly visible along microtubules (Fig. 1*E* and Movie S6). These results indicate that nanodamage alone, present in the GDP condition where lattice repair is very low, does not recruit EB1, but the GTP–tubulin incorporated at damage sites does.

### Spastin Mediated Lattice Damage and Repair Occurs In Vivo.

**Fig. 2.**
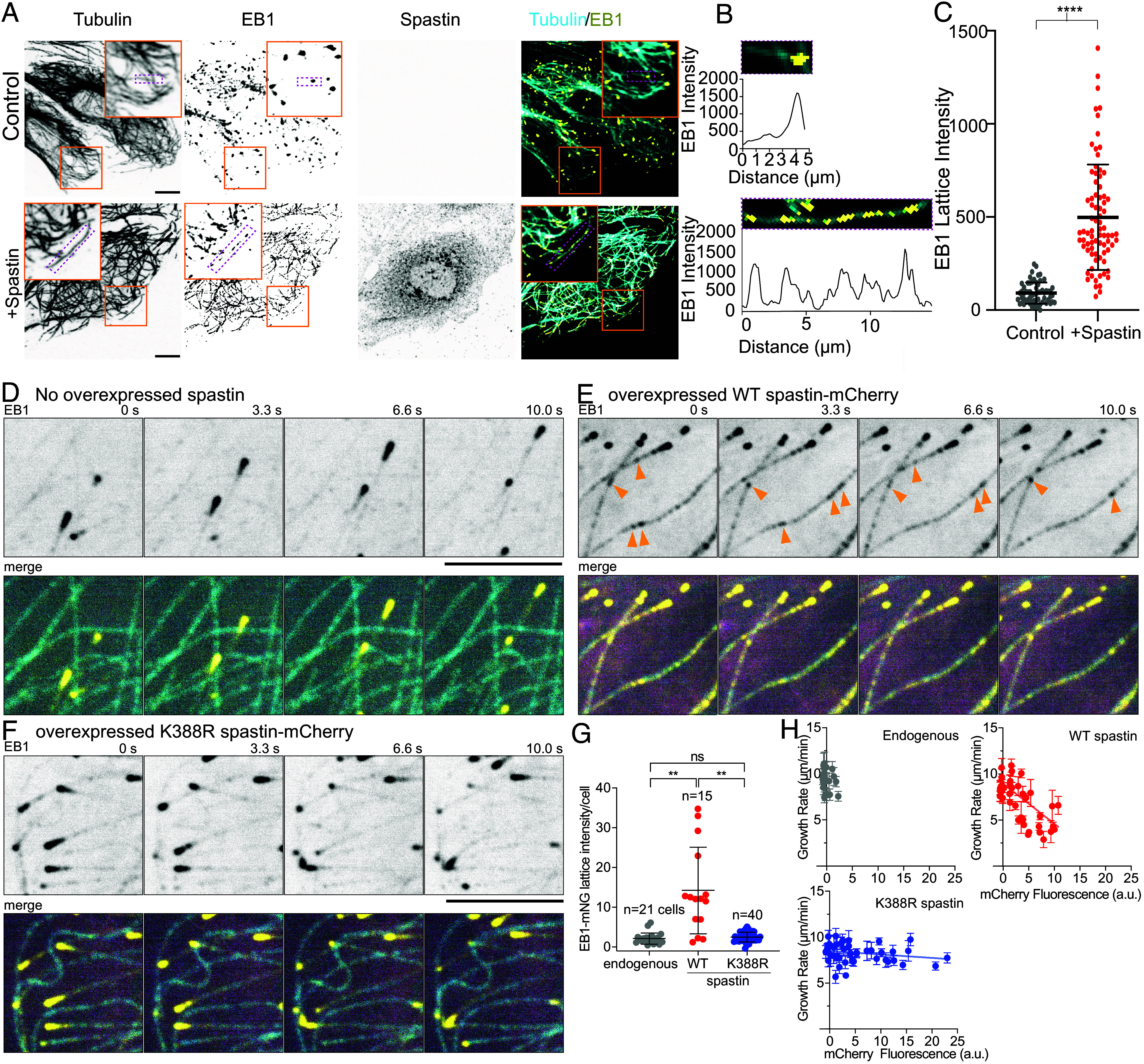
Spastin activity leads to EB1 recruitment along microtubules in cells. (*A*) Control PtK2 cells (*Top*) and cells overexpressing spastin (*Bottom*). (Scale bar, 10 µm.) (*B*) Magnification of a microtubule in the magenta box in (*A*) and the corresponding line profile showing EB1 signal at the microtubule end (*Top*) and EB1 puncta along the microtubule (*Bottom*). (*C*) EB1 mean microtubule lattice intensity in control (n = 63 microtubules from four cells) and spastin overexpressing cells (n = 79 microtubules from six cells). Error bars, SD, ****P < 0.0001 by two-tailed *t* test. (*D*–*F*) Time lapse of microtubule growth in EB1-mNG CRISPR knock-in U2OS cells without spastin overexpression, with wild-type and K388R mutant spastin-mCh overexpression. Yellow, EB1-mNG, magenta, spastin-mCh, cyan, microtubules, labeled by incubating with 500 nM SiR tubulin (*Materials and Methods*). Notably, there is weaker but sufficiently traceable SiR-tubulin signal in spastin overexpressing cells correlating with regions of increased EB1 lattice signal. This likely reflects preferential binding of the SiR-tubulin to the GDP lattice, as SiR-tubulin signal is notably weaker or absent also along EB1 comets marking the GTP–tubulin cap. Orange arrows indicate EB1 puncta along microtubules. (Scale bar, 5 µm.) See Movie S7. (*G*) Mean EB1 microtubule lattice intensity in nonexpressing (n = 21 cells), wild-type spastin-mCherry overexpressing cells (n = 15 cells) and K388R spastin-mCherry overexpressing cells (n = 40 cells); wild-type and K388R mutant expressed ~2.1- and 2.8-fold over endogenous spastin, respectively (*SI Appendix*, Fig. S2*C*); ns, *P* > 0.05, **P = 0.0029 (K388R vs. WT), **P = 0.0023 (no spastin vs. WT) by one-way ANOVA with Dunnett T3 test. See *SI Appendix*, Fig. S2*D* for experimental replicate. (*H*) Microtubule growth rates as a function of mCherry fluorescence intensity in EB1-mNG CRISPR knock-in U2OS cells and cells overexpressing wild-type or K388R spastin-mCherry. Each data point represents an average of all comets from one cell; n = 971 comets, 24 cells without spastin overexpression, 1,026 comets, 37 cells for wild-type spastin-mCherry, 1,835 comets, 43 cells for K388R spastin-mCherry, from two independent experiments each. No SiR-tubulin was used in these experiments. Error bars, 95% CI.

Next, we sought to establish whether spastin mediated nanodamage can generate GTP-islands recognized by EB1 in vivo since in cells microtubules interact with many proteins that can affect tubulin extraction by spastin and subsequent tubulin incorporation. We overexpressed spastin in *Potorous tridactylis* epithelial kidney (Ptk2) cells and analyzed endogenous EB1 localization by immunostaining. Unlike in control cells that display EB1 comets only at microtubule ends, microtubules in cells that overexpress spastin are decorated with puncta of endogenous EB1 along their lengths (Fig. 2 *A* and *B*). Consistent with this, the mean EB1 lattice intensity in cells overexpressing spastin is ~fivefold higher than in control cells (Fig. 2*C*).

We next investigated the effects of spastin overexpression in live cells by generating an EB1-mNeonGreen knock-in U2OS cell line engineered to express spastin-mCherry under a doxycycline-inducible promoter (*SI Appendix*, Fig. S2*A*). Spastin induction in these cells (~2.1-fold of endogenous levels; *SI Appendix*, Fig. S2 *B* and *C*) resulted in increased EB1 signal along microtubules with a per-cell average EB1 lattice intensity ~6.6-fold higher than nonspastin expressors (Fig. 2 *D*–*G*, *SI Appendix*, Fig. S2*D*, and Movie S7). EB1 association with the microtubule is transient (Fig. 2*E*) as would be expected because the GTP island which EB1 detects, erodes as the GTP hydrolyzes to GDP. In contrast, cells overexpressing the spastin disease mutant K388R (expression levels ~2.8-fold of endogenous spastin, *SI Appendix*, Fig. S2*C*), which binds microtubules but is inactive in severing (4) has EB1 lattice signal similar to the control (Fig. 2*G* and *SI Appendix*, Fig. S2*D*), indicating that active lattice remodeling is required for EB1 recruitment along microtubules. Interestingly, microtubule growth rates in cells overexpressing wild type spastin are repressed as a function of spastin expression levels, but not in cells expressing the inactive mutant (Fig. 2*H* and Movie S7). We speculate that this reduction in growth rates is due to the redistribution of EB1 from the microtubule ends to the lattice, and concomitant with it, the network of plus-tip trackers that promote microtubule growth. Indeed, while this work was in revision, a new study showed EB1 redistribution from microtubule ends to the rest of lattice in response to damage caused by compressive forces, accompanied by recruitment of CLASP2 to repair sites, and concomitant suppression of microtubule growth (35). In toto, these data demonstrate that, when overexpressed at low levels, spastin induces microtubule repair in cells and thus, the biochemical pathway of spastin catalyzed tubulin incorporation along the microtubule lattice discovered in our in vitro reconstitution assays is operative in the complex cellular environment.

### GTP–Tubulin Flux at Repair Sites Controls Size and Lifetime of EB1 Islands.

**Fig. 3.**
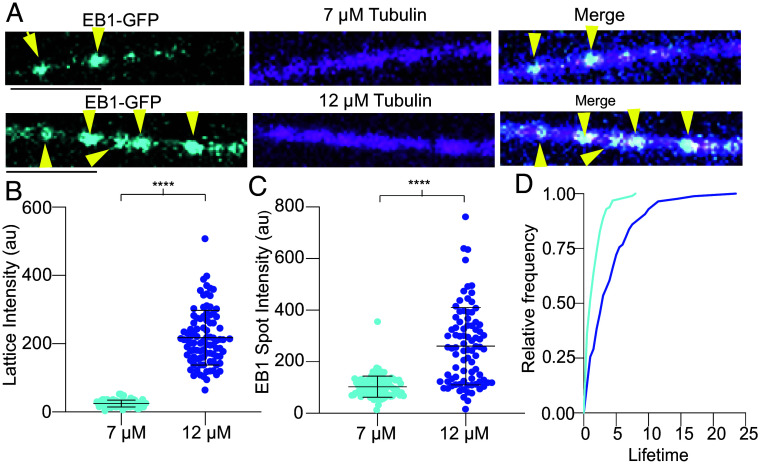
EB1-GFP island intensity and lifetimes increase with tubulin on-rate. (*A*) Images showing EB1-GFP lattice binding in the presence of spastin and 7 µM (*Top*) or 12 µM tubulin (*Bottom*). (Scale bar, 5 µm.) (*B*) Mean EB1-GFP lattice signal 10 s after spastin perfusion in the presence of 7 or 12 µM tubulin; n = 69 and 93 microtubules for the 7 and 12 µM tubulin condition, respectively from three independent experiments; ****P < 0.0001 by two-tailed *t* test. Error bars are SD. (*C*) Mean EB1-GFP puncta intensity in the presence of 20 nM spastin and 7 or 12 µM tubulin; n = 99 and 86 for the 7 and 12 µM tubulin condition, respectively; ****P < 0.0001 by two-tailed Mann-Whitney test. Error bars, SD. Puncta defined as a 7 × 7 pixel box centered on the spot with mean intensity that was at least 2.5 SD above the mean lattice intensity in the no enzyme condition. (*D*) EB1-GFP puncta cumulative probability distribution of lifetimes; n = 99 and 86 for the seven (cyan) and 12 µM tubulin (blue) condition, respectively; ****P < 0.0001 by Kolmogorov–Smirnov test.

Tubulin on-rate at microtubule ends is controlled by an array of factors (36). To determine in a simplified in vitro system whether EB1 recruitment to repair sites is influenced by the tubulin on-rate, which could potentially modulate the size of GTP–tubulin islands, we performed severing assays with dynamic microtubules in the presence of 20 nM spastin at 7 or 12 µM tubulin and EB1-GFP (Fig. 3*A*). We first pregrew microtubules to a similar average length, to ensure a similar enzyme to substrate ratio, and perfused spastin in the presence of tubulin at different concentrations. These assays showed that EB1-GFP mean lattice intensity is ~10-fold higher with 12 µM than with 7 µM tubulin, consistent with increased incorporation of GTP–tubulin at repair sites and the subsequent increased recruitment of EB1 (Fig. 3*B*). The intensity of individual EB1 puncta (spots with a mean intensity that is 2.5 SD above the background lattice signal, *Materials and Methods*) along the microtubule was also higher at 12 µM tubulin compared to 7 µM (Fig. 3*C*). This increase in intensity of EB1 puncta is also accompanied by an increase in their lifetimes. The cumulative probability reaches 0.5 in 1 s at 7 µM tubulin and 3 s at 12 µM (Fig. 3*D*), indicating that more time passes before the larger number of GTP–tubulin molecules at repair sites at the higher tubulin concentration transition to the GDP-state which is no longer recognized by EB1. We note that lifetimes of EB1 puncta in these assays do not have a simple correlation with the GTP-hydrolysis rate in the lattice after tubulin incorporation as there is continuous, active removal of tubulin by spastin, incorporation of fresh GTP–tubulin, and GTP hydrolysis as the tubulin integrates into the lattice. Spastin binds preferentially to GDP–microtubules [*SI Appendix*, Fig. S3 and as previously reported (26)], biasing tubulin extraction out of GDP- and not GTP-lattices. Thus, as the nanodamage heals with GTP–tubulin, it also has a lower binding affinity for spastin. Spastin makes multivalent interactions with the microtubule that involve one contact site between the tubulin C-terminal tail and the central pore of its AAA domain and multiple contacts between the flexible arms that emanate from the spastin hexamer (37, 38). These disordered spastin arms are good candidates for sensing microtubule lattice spacing, like microtubule associated proteins such as tau which show preference for the lattice nucleotide state (39–41).

### Tubulin GTPase Is a Timer for EB1 Island Lifetime.

**Fig. 4.**
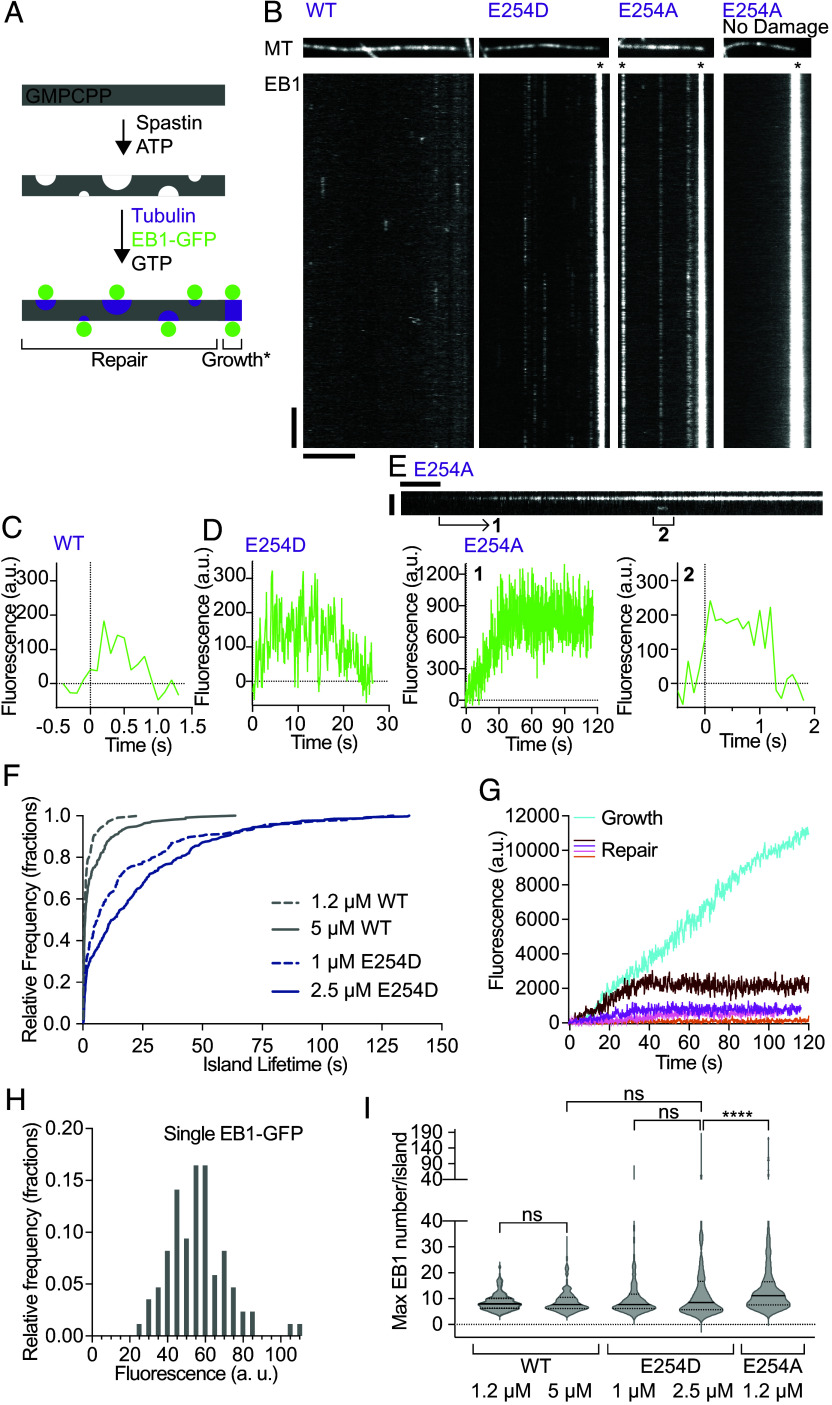
Tubulin GTP hydrolysis rate controls EB1 association with repair sites. (*A*) Experimental design schematic. GMPCPP-stabilized HiLyte647-labeled microtubules were immobilized and nanodamaged by spastin, followed by perfusion of α1A/βIII recombinant tubulin and 100 nM EB1-GFP. (*B*, *Top*) TIRF images of microtubules: undamaged or damaged and healed with 5 µM recombinant wild-type, 2.5 µM E254D or 1.2 µM E254A α1AβIII tubulin. At these concentrations microtubules have the same rates of tubulin addition at microtubule ends (13). (*Bottom*) kymographs showing EB1 binding to microtubules above. Vertical and horizontal scale bars, 5 s and 5 µm, respectively. Asterisks denote EB1 at microtubule ends. (*C* and *D*) Background-corrected fluorescence of EB1 at nanodamage sites repaired with wild-type (WT) (*C*) and E254D (*D*) tubulin as a function of time. (*E*, *Top*) kymograph with examples of long-lived (1) and short-lived (2) incorporation of E254A tubulin into the nanodamaged microtubule lattice. Vertical and horizontal scale bars, 2 µm and 5 s, respectively. (*Bottom*) Background-corrected fluorescence of EB1 as a function of time, for the two repair sites shown above. (*F*) Cumulative distribution of lifetimes of EB1-positive repair sites in the presence of wild-type or E254D tubulin; n = 214, 220, 243, and 331 repair sites for 1.2 µM wild-type, 5 µM wild-type, 1 µM E254D and 2.5 µM E254D tubulin, respectively. (*G*) Background-corrected fluorescence of EB1 as a function of time for a growing microtubule end (cyan) and repair sites (orange, magenta, brown) at 1.2 µM E254A tubulin. (*H*) Intensities of single EB1-GFP molecules immobilized to glass. (*I*) Maximal number of EB1 molecules at a repair site; n = 209, 222, 251, 345, and 185 repair sites for 1.2 µM wild-type, 5 µM wild-type, 1 µM E254D, 2.5 µM E254D, and 1.2 µM E254A tubulin, respectively. Black bars, median, 25th and 75th percentile; ns, *P* > 0.05, ****P < 0.0001 by Kruskal–Wallis test followed by Dunn’s multiple comparisons test.

To decouple the lifetime of EB1 islands from GTP-hydrolysis and the continuous extraction of tubulin by spastin, we used recombinant tubulin mutants defective in GTP-hydrolysis. We generated a tubulin mutant that is inactive in GTP hydrolysis, α-tubulin E254A, and a mutant that has a fourfold reduction in GTP-hydrolysis rate, E254D (*SI Appendix*, Fig. S4 *A* and *B*) (13, 42). E254 lies at the α-tubulin longitudinal interface and activates β-tubulin GTPase in trans when a new tubulin subunit is added (30). We performed assays in which we first incubated microtubules with spastin and ATP to generate nanodamage. To focus on repair, we limited nanodamage such that severing events were very rare. The nanodamage step was then followed by enzyme removal and subsequent perfusion of EB1-GFP and recombinant wild-type, E254A, or E254D α1AβIII tubulin (Fig. 4*A* and Movies S8–S10, *Materials and Methods*). These experiments revealed that when healed with wild-type tubulin, EB1 islands have median lifetimes of ~1 s. The lifetime increases to ~12.6 s when the slow GTP hydrolysis mutant E254D is used (Fig. 4 *B*–*F*), consistent with the tubulin GTPase rate being the timer for the EB1 island persistence. In the case of the GTP-hydrolysis defective E254A mutant, EB1 islands are peppered along the microtubule, grow over time, and eventually plateau at a maximal intensity that remains unchanged over the course of the experiment (Fig. 4 *B* and *G*). This contrasts with the EB1 intensity at the microtubule end which increases monotonically as mutant tubulin is added (Fig. 4*G*). Smaller EB1-GFP islands, containing two to three EB1-GFP molecules are frequently transient (Fig. 4 *E*, *Right*), consistent with the stochastic binding and unbinding of a small number of EB1 molecules at these sites. Such events were rare in the undamaged microtubule control (Fig. 4*B*). We calibrated the EB1-GFP signal by using single molecules immobilized to glass (Fig. 4*H*) to determine the maximum number of EB1 molecules recruited at repair sites. These measurements revealed that most islands repaired with the nonhydrolyzable E254A tubulin mutant contain ~11 EB1-GFP molecules at any one time, with islands containing as few as ~4 EB1-GFP molecules and as many as 170 (Fig. 4*I*). The large spread is likely indicative of the morphological heterogeneity of the islands. We note that islands with larger EB1-GFP signal could be due to closely spaced repair sites below the resolving power of light microscopy. We think this is an infrequent occurrence because lower enzyme concentrations did not result in robust detectable tubulin incorporation. The maximum number of EB1-GFP molecules at any one point at a repair site was lower with wild-type or the E254D mutant, consistent with the fact that some GTP–tubulin already converted to GDP as the EB1 binds (Fig. 4*I*). The maximum size of EB1 islands did not change at lower E254D tubulin concentrations, indicating that tubulin incorporation is not limiting EB1 island size under these conditions. The lifetime of EB1 islands increases with tubulin concentration, both for wild-type and the slow hydrolyzing E254D mutant (Fig. 4*F*). We speculate that this is due to the incorporation of tubulin (followed by GTP hydrolysis) from the edges of the nanodamage site where tubulin addition is more favorable because of stabilizing longitudinal and lateral interactions, as opposed to from the middle where only longitudinal interactions are initially possible. At lower tubulin on-rates the lifetime of EB1 islands may be shorter because the GTP–tubulin on rate is closer to the rate of GTP hydrolysis.

### Rescues and Depolymerization Are Sensitive to Tubulin Flux at Repair Sites.

**Fig. 5.**
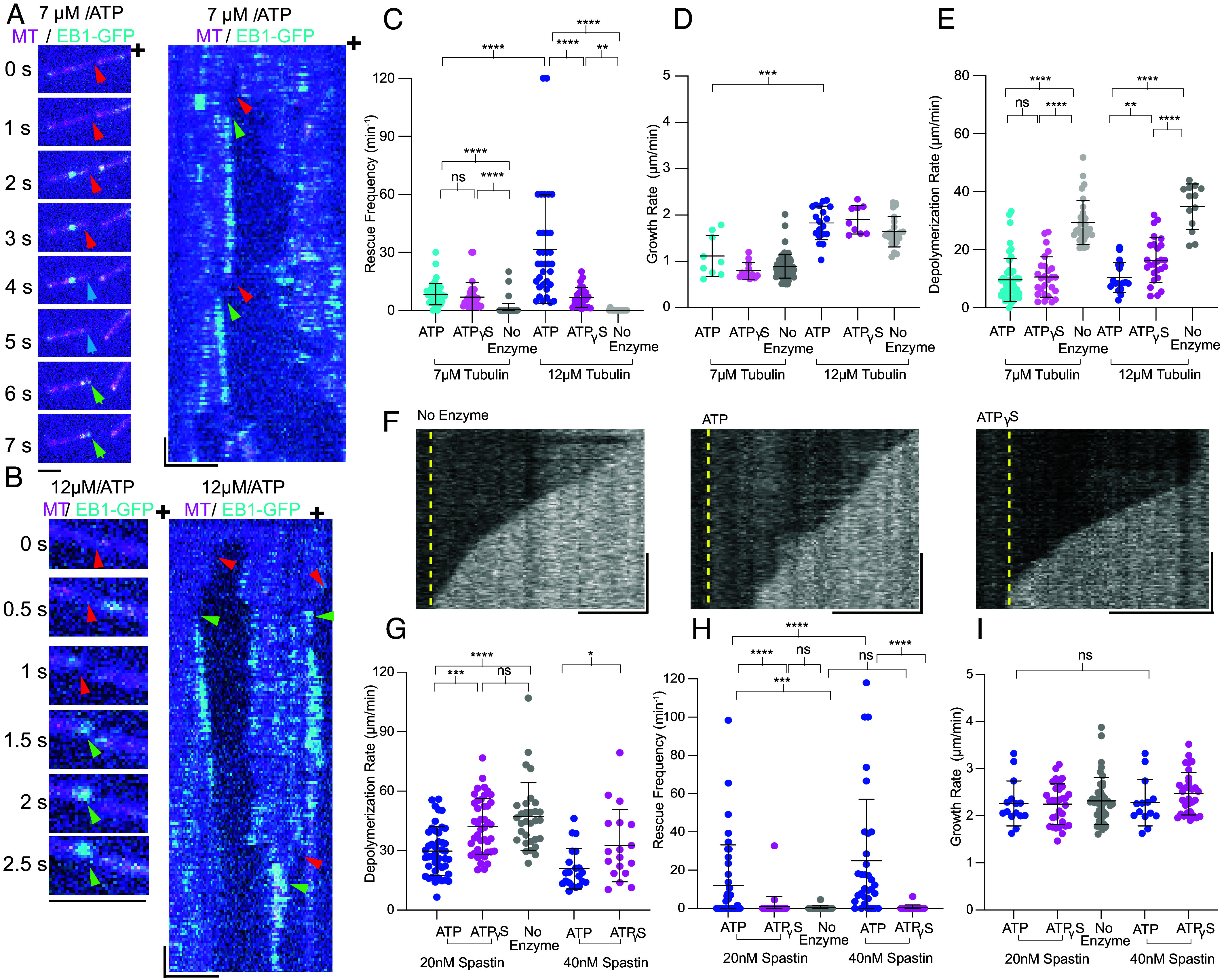
Spastin-catalyzed increase in rescues and decrease in depolymerization rates requires microtubule repair and is highly sensitive to tubulin on-rate. (*A*) Time series and kymograph of a microtubule undergoing rescue in the 7 µM tubulin, 20 nM spastin and ATP condition. [Scale bars, 2.5 µm for panels and 2.5 µm (horizontal) and 5 s (vertical) for kymograph.] We note that rescue events were scored manually, frame by frame, and not from kymographs because microtubules fluctuate as they are no longer pinned to the seed after a severing event. Depolymerization indicated with red arrows, pauses, cyan arrows, and rescues, green arrows. See Movie S11. (*B*) Time series and kymograph of a microtubule undergoing rescue in the 12 µM tubulin, 20 nM spastin, and ATP condition. [Scale bars, 2.5 µm for panels and 2.5 µm (horizontal) and 5 s (vertical) for kymograph.] Arrows defined as in *A*. See Movie S12. (*C*) Rescue frequency at 7 and 12 µM tubulin without spastin or in the presence of 20 nM spastin with ATP or ATPγS; n = 95, 47, 28, 15, 35, 26 for 7 µM tubulin with no added enzyme, spastin+ATP+7 µM tubulin, spastin+ATPγS + 7 µM tubulin, 12 µM tubulin with no added enzyme, spastin+ATP+12 µM tubulin, and spastin+ATPγS + 12 µM tubulin, respectively from two independent experiments for each condition. Error bars represent SD, ns, *P*-value > 0.05, **P = 0.002, ***P = 0.0001, ****P <0.0001 determined by the Kruskal–Wallis test and Dunnett’s test for multiple comparisons or a Mann–Whitney test for single comparisons. (*D*) Microtubule growth rates at 7 and 12 µM tubulin without spastin or in the presence of 20 nM spastin and ATP or ATPγS; n = 70, 9, 12, 24, 19, and 9 microtubules from two independent experiments in each condition for 7 µM tubulin with no added enzyme, spastin+ATP+7 µM tubulin, spastin+ATPγS + 7 µM tubulin, 12 µM tubulin no enzyme, spastin+ATP+12 µM tubulin, and spastin+ATPγS + 12 µM tubulin; error bars represent SD n.s. *P* > 0.05, ***P = 0.0009 by Welch’s *t* test. (*E*) Microtubule depolymerization rates at 7 and 12 µM tubulin without spastin or in the presence of 20 nM spastin and ATP or ATPγS; n = 30, 49, 27, 13, 18, and 26 depolymerization events from two independent experiments for 7 µM ATP and no enzyme, spastin+ATP, spastin+ATPγS, 12 µM ATP and no enzyme, spastin+ATP, spastin+ATPγS, respectively; ns, *P* > 0.05, ***P = 0.0003, ****P < 0.0001 by one-way ANOVA and Dunnett’s test for multiple comparisons. (*F*) Kymographs showing depolymerizing microtubules imaged by IRM at 12 µM tubulin in the no enzyme condition or with 20 nM spastin in the presence of ATP or ATPγS. [Scale bars, 3 s (vertical) and 2.5 µm (horizontal).] (*G*) Microtubule depolymerization rates at 12 µM tubulin without spastin or with spastin (at 20 or 40 nM) in the presence of ATP or ATPγS; n = 44, 43, 31, 21, and 18 depolymerization events from three independent experiments for 12 µM + 20 nM spastin + ATP, 20 nM spastin + ATPγS, no enzyme, 40 nM spastin + ATP and 40 nM spastin + ATPγS, respectively; ns, *P* > 0.05, *P = 0.0172, ***P = 0.0002, ****P < 0.0001 by one-way ANOVA and Kruskal–Wallis test for multiple comparisons for the 20 nM data and unpaired *t* test for the 40 nM data. (*H*) Rescue frequency at 12 µM tubulin without spastin or with spastin (at 20 or 40 nM) in the presence of ATP or ATPγS; n = 40, 39, 13, 32, and 18 from three independent experiments for 12 µM + 20 nM spastin ATP, 20 nM spastin + ATPγS, no enzyme, 40 nM spastin + ATP and 40 nM spastin + ATPγS, respectively, from. Error bars represent SD, ns, *P*-value > 0.05, ***P = 0.0010, ****P < 0.0001 determined by the Kruskal–Wallis test and Dunnett’s test for multiple comparisons or a Mann–Whitney test. For additional examples of rescues see *SI Appendix*, Fig. S7. (*I*) Microtubule growth rates at 12 µM tubulin without enzyme or with spastin (at 20 or 40 nM) in the presence of ATP or ATPγS; n = 28, 31, 42, 17, and 20 microtubules from three independent experiments for 12 µM + 20 nM spastin ATP, 20 nM spastin + ATPγS, no enzyme, 40 nM spastin + ATP and 40 nM spastin + ATPγS, respectively; ns, *P* > 0.05.

We previously reported that spastin and katanin promote rescues by catalyzing GTP–tubulin incorporation along microtubules (16). An alternative mechanism was subsequently proposed in which passive binding by spastin, and not its lattice remodeling activity, was exclusively responsible for the increase in rescues (26). This mechanism rested on the observation that rescue rates in the presence of spastin and ATP were only slightly higher than in the absence of ATP hydrolysis, although lattice repair was not monitored (26). To clarify the effect of spastin-catalyzed tubulin incorporation on microtubule dynamics we measured rescues in the presence of 20 nM spastin at 7 or 12 µM tubulin and ATP (*Materials and Methods*, Fig. 5 *A* and *B* and Movies S11 and S12). To decouple the effects of passive spastin binding from active lattice remodeling, we used ATPγS as our control. At 7 µM tubulin we observe a small, but nonsignificant increase in rescues between ATP and ATPγS (8.4 ± 0.8 min^−1^ vs. 7.0 ± 1.4 min^−1^; Fig. 5*C*). However, at 12 µM tubulin (a ~1.7-fold increase in net rate of tubulin addition at plus ends; Fig. 5*D*) we observed a ~fivefold increase in rescues with ATP compared to ATPγS (31.7 ± 4.8 min^−1^ vs. 6.8 ± 1.0 min^−1^), indicating that i) active lattice remodeling through GTP–tubulin incorporation is necessary for robust increase in rescue frequency and that ii) at the lower tubulin concentration (lower tubulin on-rate) the density of the incorporated GTP–tubulin is not sufficient to stabilize the lattice, consistent with the fact that at the lower tubulin concentration of 7 µM, EB1 islands are smaller and their lifetime is shorter than at 12 µM tubulin (Fig. 3). We could not explore the effects of higher tubulin concentrations because of de novo nucleation in the control condition under our assay conditions. The longer persistence of these GTP-islands increases the probability that they will intersect with a depolymerization event and support a rescue. Passive enzyme binding also has an effect, consistent with previous work (26) but the dramatic increase in rescues necessitates GTP–tubulin incorporation, which leads to fast rescue events (within less than 0.5 µm depolymerized length) in the ATP condition that are not observed with ATPγS. We note that rescue rates in the absence of repair (with ATPγS) do not change significantly between 7 and 12 µM tubulin. Thus, ATP-dependent lattice remodeling by spastin is required for a robust increase in rescue rates and this increase is highly sensitive to tubulin on-rate.

We also investigated the effect of tubulin on-rates on rescues by pregrowing microtubules at the same tubulin concentration, to achieve the same initial mean microtubule length, and then initiating severing and repair by perfusing 20 nM spastin together with ATP and tubulin at different concentrations. These experiments also show that rescue frequency increases with tubulin concentration (*SI Appendix*, Fig. S5*A*) while spastin concentration is kept constant, indicating that passive spastin binding is not the main driver of the observed increase in rescues. It is important to note that microtubule rescue rates, in the absence of repair, have a shallow dependence on tubulin concentration (15). In our assays with spastin we detect rescues even at 3 and 5 µM tubulin, concentrations at which rescues are not observed without microtubule repair. In conclusion, our experiments demonstrate that microtubule repair is the major contributor to the large increase in rescue frequency observed with spastin. This stabilizing effect of GTP–tubulin islands is consistent with previous work (43–47). The stabilizing effect of GTP–tubulin incorporation is also reflected in the increased stability of the newly severed plus-ends (*SI Appendix*, Fig. S5*B*). In this case also, the stability of the newly severed plus-ends varies with tubulin concentration. At 3 µM tubulin, 19% of plus-ends are stable after severing, at 5 µM, 42% and at 12 µM, 66% (*SI Appendix*, Fig. S5*B*). In contrast, classic studies using microtubule laser ablation showed that 99.3% and 91% of exposed GDP–microtubule plus-ends depolymerize even at tubulin concentrations as high as 32 and 16 µM, respectively (48, 49). Our previous study using laser ablation also showed that passive spastin binding in the presence of ATPγS does not stabilize laser-severed new plus-ends, but ATP-dependent remodeling of the microtubule does (16). The minus ends are stable regardless of tubulin concentration [*SI Appendix*, Fig. S5*C*, (16, 49)], as previously documented (48, 49).

The GMPCPP or GTP–tubulin microtubule lattice depolymerizes slower than the GDP-lattice (13, 16, 50). Therefore, we also examined the effects of GTP–tubulin incorporation on depolymerization. We found that at 7 µM tubulin, in the regime of lower tubulin incorporation, there is no significant change in depolymerization speed with spastin and ATP vs. ATPγS (Fig. 5*E*). However, at 12 µM tubulin microtubules depolymerize ~50% slower in the ATP condition compared to ATPγS (10.4 vs. 16.5 µm/min; Fig. 5*E*). Passive binding also slows depolymerization (apo vs. ATPγS comparison), consistent with previous work (26), but lattice remodeling slows depolymerization further. The presence of 50 nM EB1-GFP as a readout of repair does not affect rescue or depolymerization rates in these assays (*SI Appendix*, Fig. S6 *A*–*D*). Thus, GTP–tubulin islands catalyzed by spastin slow depolymerization speed.

To further cement our results, we examined the effect of spastin on microtubule dynamics at higher frame rates using IRM (Fig. 5 *F*–*I* and *SI Appendix*, Fig. S7), and unlabeled tubulin to exclude any confounding effects due to fluorophores which are known to affect tubulin behavior. As observed with TIRF, the IRM assays show that at 20 nM spastin with ATP, microtubules depolymerize slower than with ATPγS (29.8 vs. 42.3 µm/min, Fig. 5 *F* and *G*). Notably, unlike in the assays with fluorescent tubulin, passive enzyme binding does not have a significant effect on depolymerization speed. This may reflect an increased binding of the enzyme to fluorescently labeled microtubules compared to unlabeled ones. The decrease in depolymerization speed between the ATP and ATPγS condition is also visible at 40 nM spastin but is more muted than at 20 nM, likely because of the increased number of spastin molecules bound to the microtubule at this higher concentration. Notably, the higher enzyme concentration does not increase rescue rates with ATPγS, indicating that while passive enzyme binding contributes to reducing depolymerization speed, it does not contribute significantly to the observed increase in rescues (Fig. 5*H*). Consistent with our TIRF data, and with a model in which enzyme-dependent lattice remodeling promotes rescues, rescue rates in the spastin ATP condition are more than an order of magnitude higher than with ATPγS, both at 20 and 40 nM spastin (12.2 vs. 1.0 min^−1^ and 24.9 vs. 0.4 min^−1^, respectively; Fig. 5*H*).

In conclusion, our data from the TIRFM- and IRM assays at different tubulin and enzyme concentrations demonstrate the importance of enzyme-catalyzed lattice repair in promoting microtubule rescue and the sensitivity of the increase in rescues to tubulin concentration. These results are consistent with our previous work that showed that microtubules peppered with spastin-catalyzed repair sites rescue quickly, while microtubules without repair and only bound to inactive spastin do not (16).

## Discussion

Until recently, GTP–tubulin was thought to be exclusively present at growing microtubule ends, where it forms a stabilizing GTP cap (14, 51). This view was challenged when studies using an antibody sensitive to the GTP–tubulin conformation reported that in cells, microtubules contain GTP–tubulin islands peppered throughout their lengths and to a lesser extent when microtubules are polymerized from purified tubulin in vitro (43). These GTP–tubulin islands were proposed to be remnants of a polymerization process that did not completely convert the incorporated GTP- tubulin into the GDP-bound state. We now know that GTP–tubulin islands are incorporated into preexisting microtubules through a repair process catalyzed by mechanical damage (35, 47, 52, 53), severing enzymes (16), kinesin and dynein motors (46, 54, 55), and drug-induced lattice defects (45). Cryo-tomography also revealed microtubules with lattice defects in neurons and fibroblasts (56–59). This recent body of work points toward a much larger dynamicity of the microtubule lattice than previously appreciated, mediated by mechanical and biochemical signals (60).

Our work demonstrates that the large increase in rescues induced by spastin requires incorporation of GTP–tubulin (i.e., repair) and is highly sensitive to GTP–tubulin concentration (Fig. 5). At low tubulin concentrations, there is less net accumulation of GTP–tubulin in an island, likely because the GTP-hydrolysis rate is comparable to that of tubulin incorporation (Figs. 3 and 4). Under these conditions, large enough numbers of GTP–tubulin molecules are unlikely to accumulate at repair sites and therefore the effect on rescues is smaller (Fig. 5). At higher tubulin concentrations, the incorporation rate will exceed that of hydrolysis, leading to larger GTP–tubulin islands that take longer to erode (Fig. 3) and result in a large increase in rescue rates. The steepness of this response shows that a small quantitative change in tubulin on-rate, which can be modulated by effectors, can lead to a qualitative difference in microtubule dynamics. In addition, the net flux of GTP–tubulin into the microtubule is also a function of the rate of extraction i.e., severing enzyme concentration; when this is higher, so is the rescue rate (Fig. 5). The dependence of rescues on these three rates: tubulin extraction, GTP–tubulin incorporation, and tubulin GTPase rate (16) makes this a highly tunable process that enables severing enzymes to be versatile tools to either destroy or amplify microtubule arrays depending on cellular context. In addition to shedding light on the mechanism of microtubule dynamics regulation by severing enzymes, our experiments are also a fundamental characterization of the repair process, relevant more broadly for other forms of lattice induced damage.

Our demonstration that GTP–tubulin incorporation along the microtubule increases rescue rates is consistent with recent work that shows that microtubules repaired after mechanical, motor, light- or drug-induced damage, also rescue at higher frequencies (44, 47, 61) and that rescues occur preferentially at repair sites (44, 45). Our data do not support the alternative mechanism proposed by Kuo et al. (26) whereby passive enzyme binding is solely responsible for the observed increase in rescues, with no contribution from repair. In that study tubulin lattice incorporation was not monitored and therefore it is not possible to know in what regime of tubulin incorporation the experiments were conducted. We also note that microtubule growth rate at 12 µM tubulin in that study is ~2.6 times lower than in our assays (1.8 ± 0.3 vs. 0.7 ± 0.12 µm/min), corresponding to a regime of low tubulin incorporation into the lattice (comparable to the 7 µM tubulin condition in this study) where lattice repair makes a smaller contribution to rescue frequency as our work shows and where our studies are in agreement. The Kuo et al. study (26) also used rigor kinesins or an antitubulin antibody to pin down microtubules to the glass to facilitate monitoring of microtubules. The stabilizing effect of the rigor kinesins or antibodies bound along the microtubule might mask the effects of lattice tubulin incorporation on rescues, thus dampening the effect of repair. Overall, our in-depth analysis of the repair process cements the important contribution of GTP–tubulin incorporation through nanodamage and repair to microtubule rescues.

EBs recognizes GTP–tubulin (13, 32–34) and sit at the nexus of a large network of proteins recruited to microtubule ends to regulate their dynamics (62, 63). Our work shows that EB1 binding along the microtubule in the presence of spastin is due to the recognition of newly incorporated GTP–tubulin at the damage site and not recognition of damage alone, as proposed (25). We speculate that this discrepancy might be because previous experiments (25) were conducted with microtubules with artificially generated open lattices and defects that are much larger than the nanodamage introduced physiologically by severing enzymes. Finally, we show that the repair observed in vitro is also operative in cells where lattice remodeling by overexpressed spastin (~2.1-fold above endogenous level) leads to redistribution of EB1 from the tips to repair sites along microtubules. Thus, our work demonstrates that repair is an obligatory step in severing enzyme reactions both in vitro and in vivo. A recent study using injection of fluorescent tubulin in cells showed incorporation of tubulin along microtubules (64), likely due to a combination of factors ranging from mechanical forces to microtubule effectors. The EB1 recruited to GTP–tubulin islands in cells is part of a larger network of MAPs traditionally associated with microtubule ends. Indeed, previous work showed recruitment of CLIP-170 to EB1 islands (47), and the recruitment of CLIP-170 and p150-Glued to EB1 islands when CLASPs are depleted (65). More recently, CLASP2 was shown to localize to EB1 islands generated by compressive forces (35). Some of these MAPs could increase the rate of tubulin incorporation at the nanodamage sites (45). Others, like depolymerizing kinesins could enlarge the initial nanodamage to depolymerize stable microtubule structures, such as bundles, more efficiently. Spastin, has been implicated in the dissolution of the stable microtubule bundles at the cytokinetic bridge (66). Some MAPs can also recognize the damaged lattice, as shown for SSNA1 (67) and protect it against depolymerization. Moreover, these repair sites could also be used as platforms for crosstalk with other cytoskeletal polymer networks as CLIP-170 interacts with the formin mDia to initiate actin polymerization from microtubule ends (68). Identifying the effectors recruited at repair sites and how they modulate the kinetics of repair or depolymerization from the damage site, as well as their crosstalk with other cytoskeletal filament systems will be an interesting area of exploration.

## Materials and Methods

Due to space constraints, details for protein expression and purification are provided in the *SI Appendix* as well as complete experimental details for all experiments. Statistical analyses were performed in Prism (GraphPad Inc.) and are indicated in figure legends.

## Supplementary Material

Appendix 01 (PDF)

Movie S1. TIRFM acquired movie of GMPCPP-capped GDP-microtubules (magenta) in the presence of 10 nM spastin and 12 μM GDP-tubulin (cyan). Tubulin was perfused first, followed by tubulin together with spastin. Yellow arrows indicate tubulin incorporation.

Movie S2. TIRFM acquired movie of GMPCPP-capped GDP-microtubules (magenta) in the presence of 10 nM spastin and 12 μM GTP-tubulin (cyan). Tubulin was perfused first, followed by tubulin together with spastin. Yellow arrows indicate tubulin incorporation.

Movie S3. TIRFM acquired movie of GMPCPP-capped GDP-microtubules (magenta) in the presence of 10 nM spastin and 7 μM GDP-tubulin (cyan). Tubulin was perfused first, followed by tubulin together with spastin.

Movie S4. TIRFM acquired movie of GMPCPP-capped GDP-microtubules (magenta) in the presence of 10 nM spastin and 7 μM GTP-tubulin (cyan). Tubulin was perfused first, followed by tubulin together with spastin. Yellow arrows indicate tubulin incorporation.

Movie S5. TIRFM acquired movie of GMPCPP-capped GDP-microtubules (magenta) in the presence of 5 nM spastin and 12μM GDP-tubulin and EB1-GFP (cyan).

Movie S6. TIRFM acquired movie of GMPCPP-capped GDP-microtubules (magenta) in the presence of 5 nM spastin and 12μM GTP-tubulin and EB1-GFP (cyan). Yellow arrows show EB1 recruitment along the microtubule.

Movie S7. Microtubules in EB1-mNG CRISPR knock-in U2OS cells without spastin overexpression, with wild-type spastin-mCherry overexpression (2.1-fold over endogenous) or K388R mutant spastin-mCh overexpression (2.8-fold over endogenous). Yellow, EB1-mNG, magenta, spastin-mCherry, cyan, microtubules.

Movie S8. TIRFM movie of EB1 recruitment to nanodamaged microtubules repaired with wild-type recombinant tubulin. EB1 marked by magenta arrows.

Movie S9. TIRFM movie of EB1 recruitment to nanodamaged microtubules repaired with E254D mutant recombinant tubulin. EB1 along the microtubule marked by magenta arrows, EB1 at microtubule ends marked by cyan arrows.

Movie S10. TIRFM movie of EB1 recruitment to nanodamaged microtubules healed with E254A mutant recombinant tubulin. EB1 along the microtubule marked by magenta arrows, EB1 at microtubule ends marked by cyan arrows.

Movie S11. TIRFM acquired microtubule dynamics at 7 μM tubulin and 20 nM spastin in the presence of 1mM ATP and 50 nM EB1-GFP. Microtubules (magenta), EB1 (cyan). Depolymerization indicated by red arrows, pauses by cyan arrows and rescues by green arrows.

Movie S12. TIRFM acquired microtubule dynamics at 12 μM tubulin and 20 nM spastin in the presence of 1mM ATP and 50 nM EB1-GFP. Microtubules (magenta), EB1 (cyan). Depolymerization indicated by red arrows, pauses by cyan arrows and rescues by green arrows.

## Data Availability

Plasmids used in this study are available on Addgene or from the corresponding author. Custom data analysis macros were published previously (69) and are deposited at https://github.com/RollmecakLab (70). Source data for figures and IRM movie are deposited on Figshare at 10.6084/m9.figshare.32091340 (71).
