# Supplementary material for: Tubulin flux at spastin-induced nanodamage sites regulates microtubule rescue frequency and EB1 lifetimes
Source: Proc Natl Acad Sci U S A. 2026 May 26;123(22):e2517683123. doi: 10.1073/pnas.2517683123 (PMC13229199; doi:10.1073/pnas.2517683123)
Supplement: Supplementary file 1 — Appendix 01 (PDF) [file pnas.2517683123.sapp.pdf]

PNAS

Supporting Information for

Tubulin flux at spastin-induced nanodamage sites regulates microtubule rescue frequency and EB1 lifetimes

Ewa Szczesna [a,*], Jeffrey O. Spector [a,*], Stephanie L. Sarbanes [a], Jiayi Chen [a], Agnieszka Szyk and Antonina Roll-Mecak [a,b,§]

Antonina Roll-Mecak
Email: Antonina@mail.nih.gov

This PDF file includes:

Figures S1 to S7
Legends for Movies S1 to S12
Extended Materials and Methods
SI References

Other supporting materials for this manuscript include the following:

Movies S1 to S12

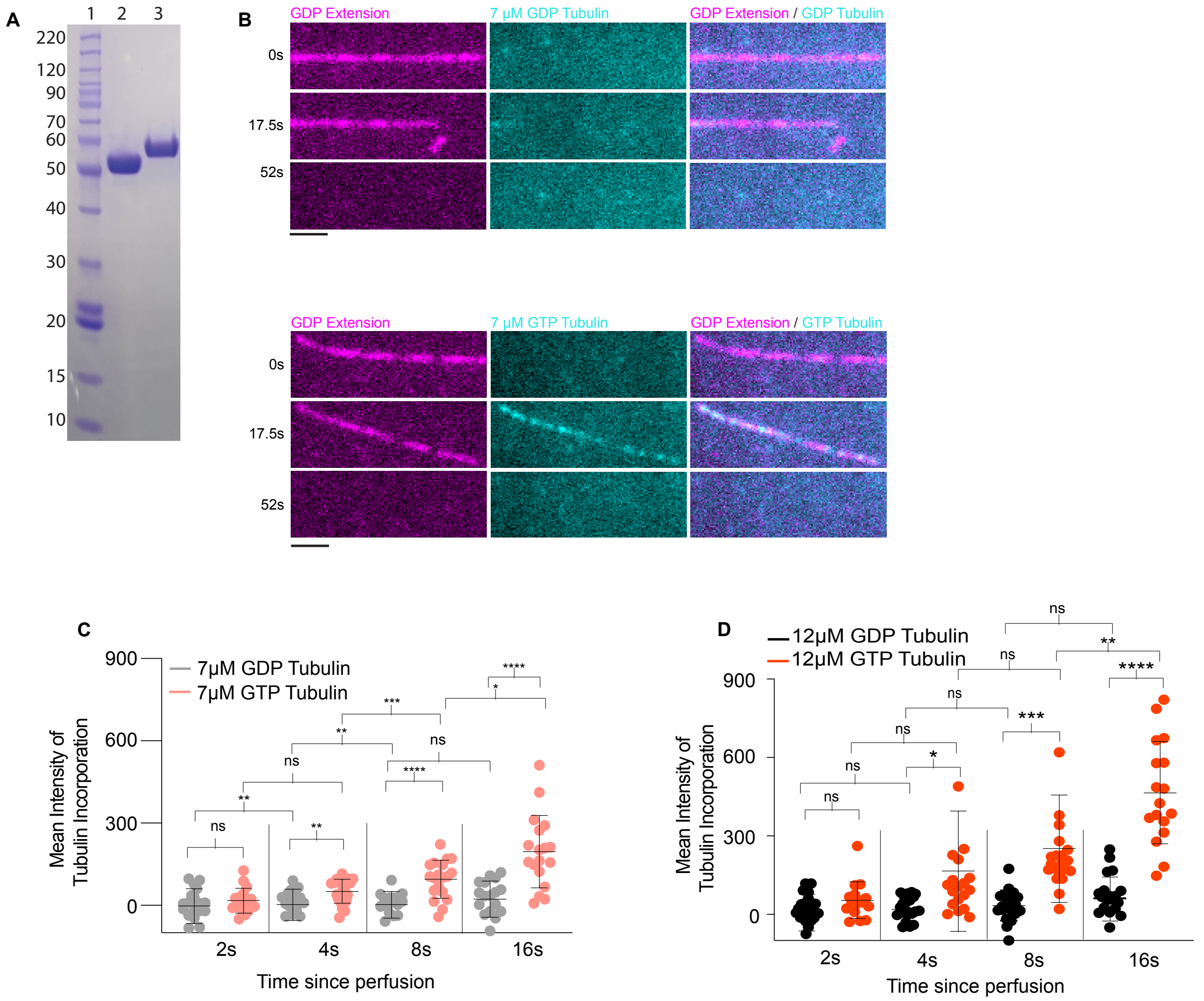

Figure S1. GTP-tubulin, not GDP-tubulin incorporates efficiently into spastin nano-damaged microtubules.

A) SDS-PAGE gel of purified proteins. Lane (1): BenchMark Protein Ladder 5 µl – ThermoFisher Catalog number 10747012, (2): Spastin 2 µg, (3) EB1-GFP 2 µg

B) Representative time series of GMPCPP-capped GDP-microtubule extension (magenta) in the presence of 10 nM spastin and 7 µM GDP-tubulin (top) or GTP-tubulin (bottom) (cyan); scale bar, 2 µm.

C) Background-corrected mean intensity of tubulin incorporation into GMPCPP-capped GDP-microtubule extensions at 7 µM tubulin in the presence of spastin and ATP. For t = 2s, n = 19, 17 microtubules for GDP and GTP, respectively. For GDP at 2s *vs*. 4s **, p = 0.0079. For t = 4s, n = 19, 22 microtubules for GDP and GTP respectively, ** p = 0.0086. For GDP at 4s *vs*. GDP at 8s, ** p = 0.0042. For GTP at 4s vs. 8s; *** p = 0.001. For 8s n = 18, 19 for GDP and GTP respectively, **** p < 0.0001. For GTP at 8s *vs*. GTP at 16s, * p = 0.0159. For 16s, n = 18, 19 microtubules for GDP and GTP respectively, **** p < 0.0001 by one-way ANOVA with Dunnett T3 test for multiple comparisons.

D) Background-corrected mean intensity of tubulin incorporation as in C, but at 12 µM soluble tubulin. For t = 2s, n = 25, 16 microtubules for GDP and GTP respectively. For t = 4s, n = 21, 19 microtubules for GDP and GTP respectively, * p = 0.0026. For t = 8s, n = 20, 22 microtubules for GDP and GTP respectively, *** p = 0.0001. For GTP at 8s vs. 16s **, p = 0.0043. For t = 16s, n = 20, 17 microtubules for GDP and GTP respectively, **** p < 0.0001 by one-way ANOVA with Dunnett T3 test to correct for multiple comparisons.

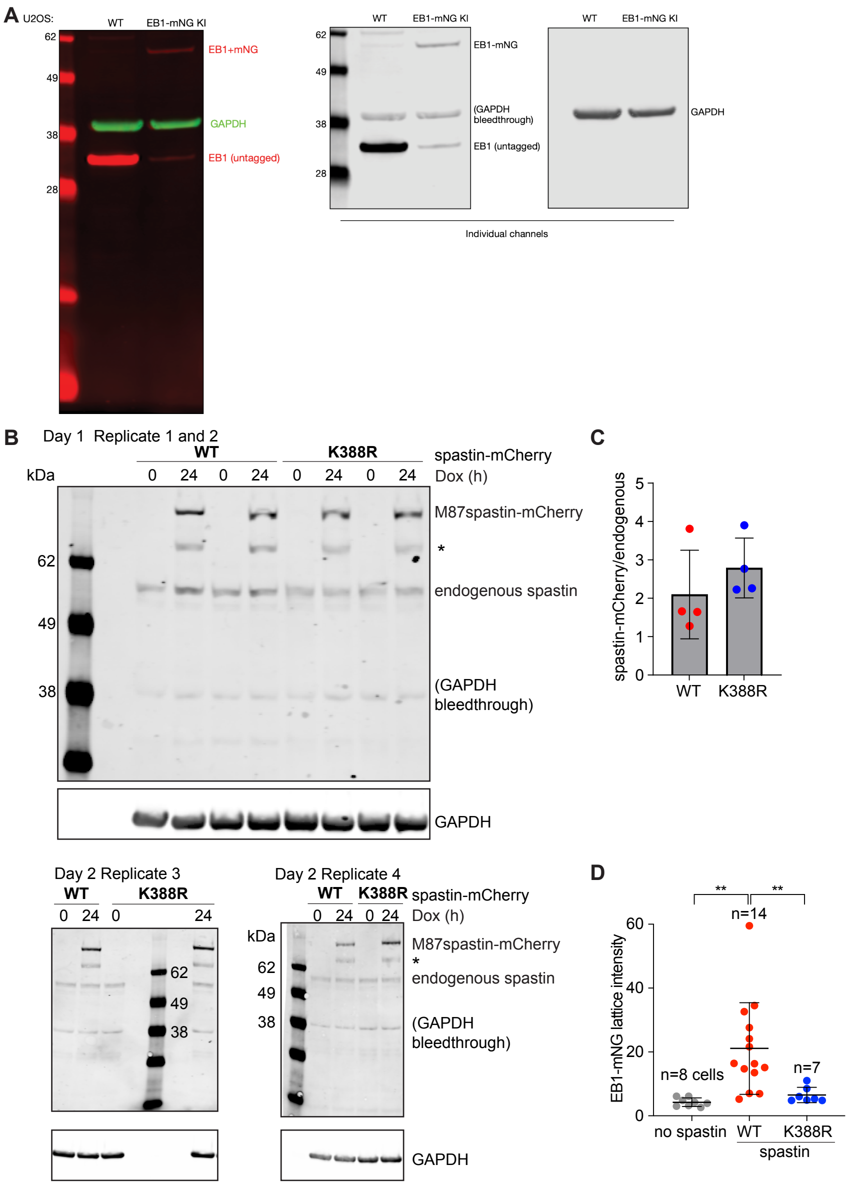

Figure S2. Validation of EB1-mNeonGreen CRISPR Knock-in U2OS line and inducible spastin cell lines.

A) Validation of EB1-mNeonGreen CRISPR Knock-in. Western blot of parental wild type (WT) and selected EB1-mNG knock-in (predicted size untagged=31 kDa, tagged=58 kDa) clone probed with anti-EB1 (N-terminal) antibody confirms clone is heterozygous for tagged EB1. GAPDH used as a loading control.

B) Validation of Dox-inducible spastin cell line. Western blots of EB1-mNG knock-in cells with addition of dox-inducible WT or K388R spastin-mCherry construct probed with anti-spastin antibody confirms dox-inducible expression (predicted size = 85 kDa) of endogenous expression (predicted size = 58 kDa) for WT and K388R spastin-mCherry, respectively, at 24 hours post-induction (* denotes degradation products). GAPDH, loading control.

C) Quantification of overexpressed spastin-mCherry wild type and K388R constructs. n = 4 experiments run on three gels.

D) Per cell mean EB1 microtubule lattice intensity in control cells (n = 8 cells) and spastin-overexpressing cells (n = 14 and 7 cells for WT and K388R spastin-mCherry, respectively); **, p = 0.0019 (no spastin *vs.*

wild type spastin), **, p = 0.0093 (wild-type spastin *vs*. K388R spastin) by one-way ANOVA with Dunnett T3 test to correct for multiple comparisons. The absolute EB1 intensity in these experiments is different from that shown in Figure 2G because of a change in microscope and cameras between these two experiments due to a flood that destroyed out instruments.

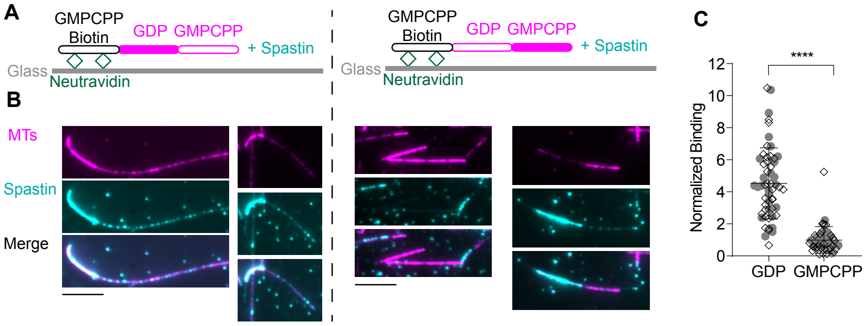

Figure S3. Spastin prefers the GDP microtubule lattice.
A) Schematic of experimental setup. Microtubules with GDP and GMPCPP segments labeled with different percentages of HiLyte647-tubulin were immobilized on glass *via* biotinylated, unlabeled microtubule seeds and Atto488-labeled spastin with ATPγS was perfused into the chamber (Materials and Methods). Left, GDP sections, 20% labeled, GMPCPP sections, 5% labeled with HiLyte647-tubulin; Right, GDP sections, 5% labeled, GMPCPP, 20% labeled with HiLyte647-tubulin.
B) Left, spastin (cyan), GDP-microtubule segment (bright magenta) and GMPCPP-microtubule segment (dim magenta). Right, spastin (cyan), GDP-microtubule segment (light magenta) and GMPCPP-microtubule segment (bright magenta). Scale bar, 5 µm.
C) Background-corrected intensity normalized to GMPCPP segments. Line, mean; error bars, S.D; n = 56 and 48 microtubule segments for GDP and GMPCPP, respectively from 8 independent experiments performed on two different days. ****, $p < 0.0001$ by two-tailed t-test. Grey circles are for data points corresponding to experimental scheme on the left in panel A, diamonds correspond to data points corresponding to experimental scheme on the right in panel A.

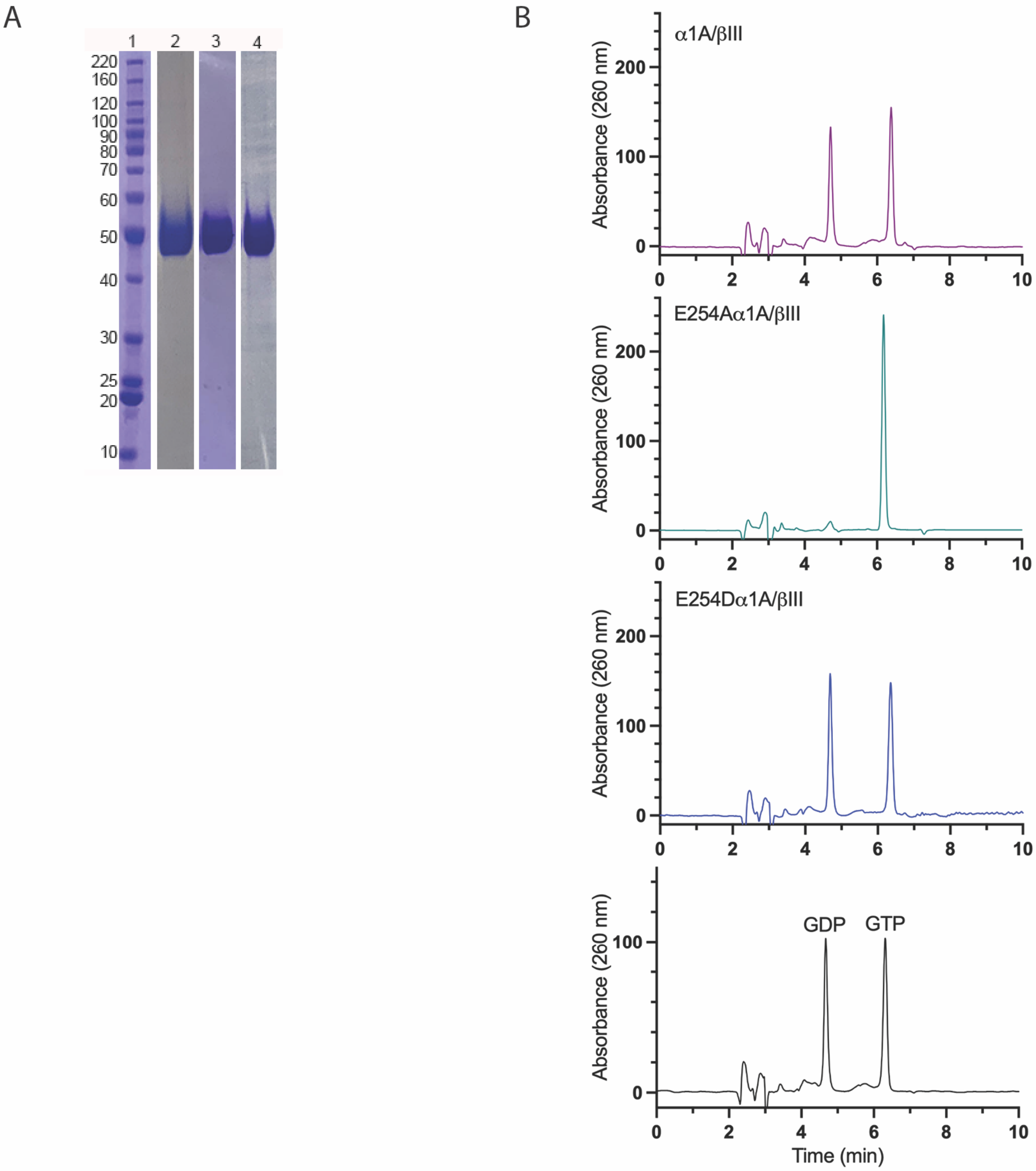

Figure S4. Analysis of recombinant tubulin mutants.

A) Coomassie blue-stained SDS–PAGE gel showing 4 μg purified recombinant WT (lane 2), E254D (lane 3) and E254A (lane 4) α1A/βIII tubulin constructs.

B) HPLC chromatograms of nucleotides extracted from microtubules polymerized from recombinant WT, E254A and E254D α1A/βIII tubulin with comparison to GDP and GTP standards (Materials and Methods). Note that the time resolution of the assay is not fast enough to detect the lower GTPase rate for the E254D mutant. This was obtained from the comet data in Roostalu *et al.*, 2020.

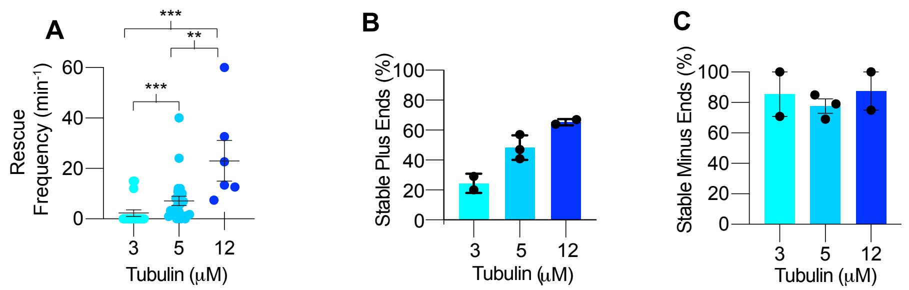

Figure S5. Spastin-catalyzed increase in microtubule rescue frequency and the stability of newly severed plus-ends is sensitive to tubulin on-rates.

A) Rescue frequency at 3, 5 and 12 μM tubulin; n = 18, 24 and 6 microtubules from 2, 3, and 2 experiments; Error bars, S.E.M., ** $p < 0.01$, *** $p < 0.001$, by a two-tailed Mann-Whitney test. The small number of events in the 12 μM condition was due to many events rescuing faster than the frame rate.

B) Percentage of observed stable plus ends at 3, 5 and 12 μM tubulin; n = 2, 3 and 2 experiments with 39, 34 and 26 severing events analyzed for the 3, 5 and 12 μM tubulin conditions. Error bars, S.D.

C) Percentage of observed stable minus ends at 3, 5 and 12 μM tubulin; n = 2, 3, and 2 experiments with 34, 31 and 20 microtubules analyzed for 3, 5 and 12 μM tubulin condition. Error bars, S.E.M.

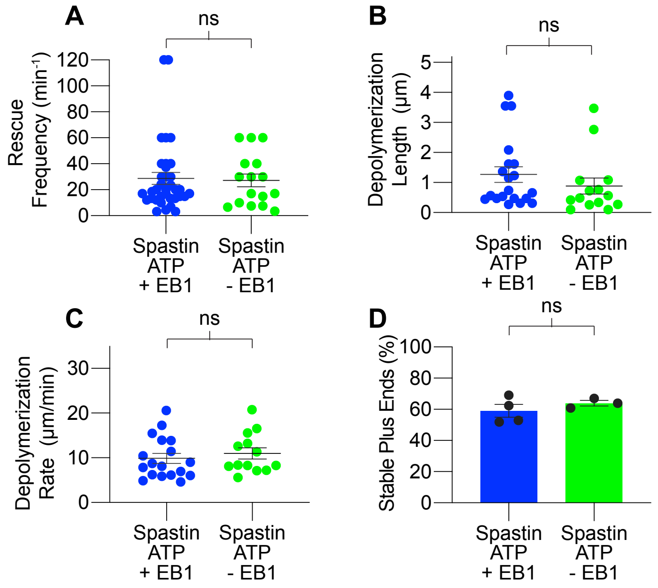

Figure S6. The addition of 50 nM EB1-GFP does not affect microtubule dynamics parameters.

A) Rescue rates with and without EB1-GFP; n = 35 and 16 microtubules, respectively; n= 4 and 3 experiments with and without EB1-GFP, respectively.

B) Depolymerization lengths with and without EB1-GFP; n = 20 and 14 microtubules, respectively; n = 4 and 3 experiments with and without EB1-GFP, respectively.

C) Depolymerization rate with or without EB1-GFP; n = 18 and 13 microtubules, respectively; ns, $p > 0.05$ by Mann-Whitney test. n= 4 and 3 experiments with and without EB1-GFP, respectively.

D) Stability of newly severed microtubule plus-ends with or without EB1-GFP; n= 4 and 3 experiments with and without EB1-GFP, respectively. Error bars, S.E.M.

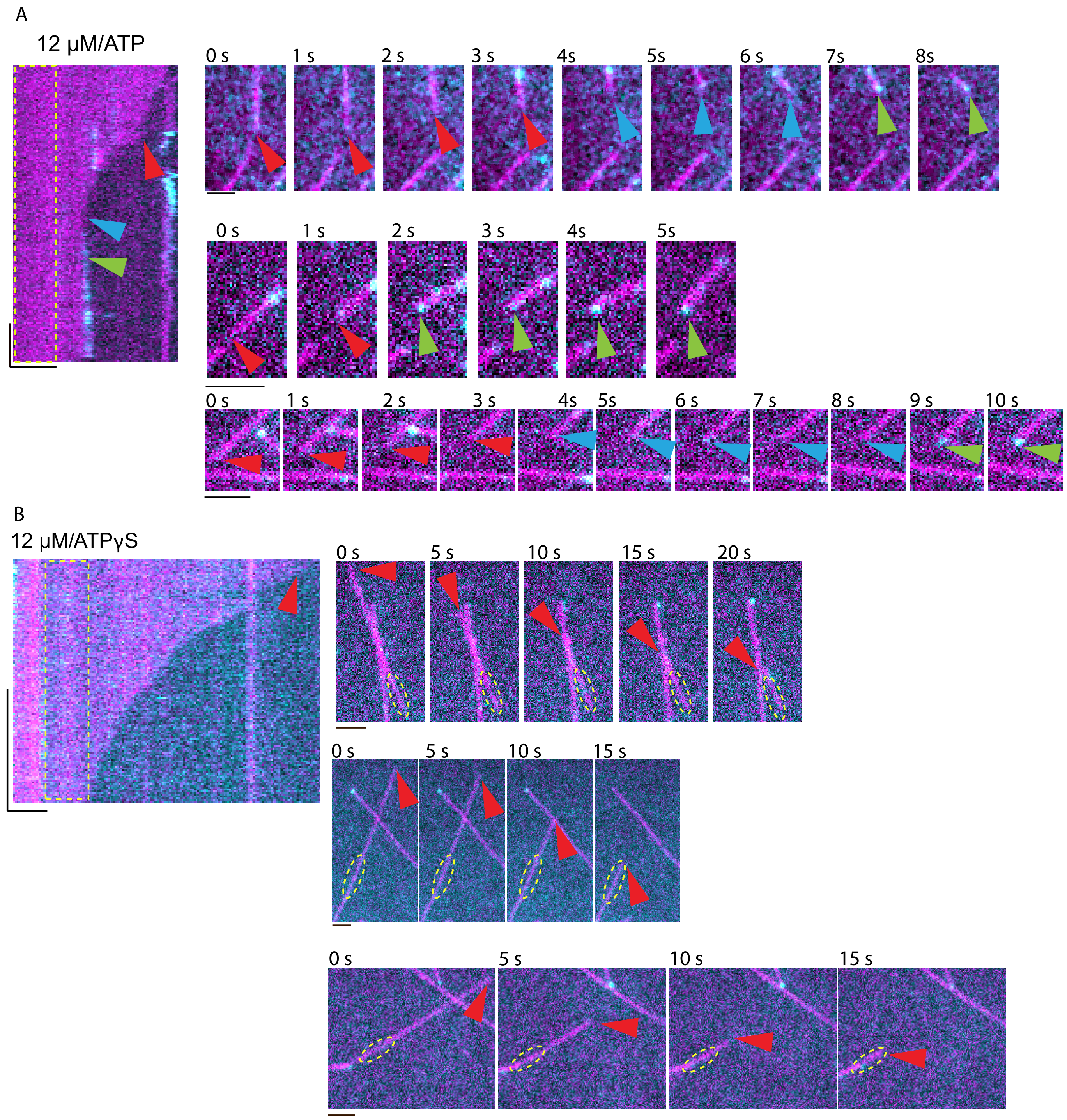

Figure S7. Examples of microtubule rescue events.

A) Left, Kymograph of a microtubule undergoing rescue in the 12μM tubulin, 20 nM spastin with ATP condition recorded by IRM. Scale bars, 2.5μm for panels and 2.5μm (horizontal) and 5s (vertical) for kymograph. We note that rescue events were scored manually, frame by frame, and not from kymographs because microtubules fluctuate (as most are no longer pinned to the seed after a severing event). Right, time series showing examples of rescue events. Depolymerization indicated with red arrows, pauses with cyan arrows and rescues with green arrows.

B) Left, Kymograph of a microtubule depolymerizing in the 12μM tubulin, 20 nM spastin and ATPγS condition recorded by IRM. Right, time series showing examples of depolymerization events. Dashed ovals

delineate the GMPCPP seed. Red arrows mark a depolymerization event. Scale bars, 2.5µm (horizontal) and 5s (vertical) for kymograph and 2.5µm for panels.

Movie S1
TIRFM acquired movie of GMPCPP-capped GDP-microtubules (magenta) in the presence of 10 nM spastin and 12 µM GDP-tubulin (cyan). Tubulin was perfused first, followed by tubulin together with spastin. Yellow arrows indicate tubulin incorporation.

Movie S2
TIRFM acquired movie of GMPCPP-capped GDP-microtubules (magenta) in the presence of 10 nM spastin and 12 µM GTP-tubulin (cyan). Tubulin was perfused first, followed by tubulin together with spastin. Yellow arrows indicate tubulin incorporation.

Movie S3
TIRFM acquired movie of GMPCPP-capped GDP-microtubules (magenta) in the presence of 10 nM spastin and 7 µM GDP-tubulin (cyan). Tubulin was perfused first, followed by tubulin together with spastin.

Movie S4
TIRFM acquired movie of GMPCPP-capped GDP-microtubules (magenta) in the presence of 10 nM spastin and 7 µM GTP-tubulin (cyan). Tubulin was perfused first, followed by tubulin together with spastin. Yellow arrows indicate tubulin incorporation.

Movie S5
TIRFM acquired movie of GMPCPP-capped GDP-microtubules (magenta) in the presence of 5 nM spastin and 12µM GDP-tubulin and EB1-GFP (cyan).

Movie S6
TIRFM acquired movie of GMPCPP-capped GDP-microtubules (magenta) in the presence of 5 nM spastin and 12µM GTP-tubulin and EB1-GFP (cyan). Yellow arrows show EB1 recruitment along the microtubule.

Movie S7
Microtubules in EB1-mNG CRISPR knock-in U2OS cells without spastin overexpression, with wild-type spastin-mCherry overexpression (2.1-fold over endogenous) or K388R mutant spastin-mCh overexpression (2.8-fold over endogenous). Yellow, EB1-mNG, magenta, spastin-mCherry, cyan, microtubules

Movie S8
TIRFM movie of EB1 recruitment to nanodamaged microtubules repaired with wild-type recombinant tubulin. EB1 marked by magenta arrows.

Movie S9
TIRFM movie of EB1 recruitment to nanodamaged microtubules repaired with E254D mutant recombinant tubulin. EB1 along the microtubule marked by magenta arrows, EB1 at microtubule ends marked by cyan arrows.

Movie S10
TIRFM movie of EB1 recruitment to nanodamaged microtubules healed with E254A mutant recombinant tubulin. EB1 along the microtubule marked by magenta arrows, EB1 at microtubule ends marked by cyan arrows.

Movie S11

TIRFM acquired microtubule dynamics at 7 μM tubulin and 20 nM spastin in the presence of 1mM ATP and 50 nM EB1-GFP. Microtubules (magenta), EB1 (cyan). Depolymerization indicated by red arrows, pauses by cyan arrows and rescues by green arrows.

Movie S12
TIRFM acquired microtubule dynamics at 12 μM tubulin and 20 nM spastin in the presence of 1mM ATP and 50 nM EB1-GFP. Microtubules (magenta), EB1 (cyan). Depolymerization indicated by red arrows, pauses by cyan arrows and rescues by green arrows.

Supplementary Materials and Methods

Protein expression and purification

Drosophila melanogaster spastin (209-C terminus) was purified by affinity chromatography and ion exchange as previously described (1). For the microtubule binding experiments, spastin was labeled with a fluorescent peptide (Atto488-LPETGG-COOH) using Sortase A (2). A Tev cleavage site (ENLYFQ/GGG) was added to the N-terminus of spastin, leaving the N-terminal tag GGG for labeling. The labeled protein was purified on a Superose 6 10/300 GL size exclusion column (GE Healthcare) to remove the unreacted peptide. The labeling efficiency was 66% and the labeled enzyme is active in severing assays. Full-length *homo sapiens* EB1 fused to GFP at the C-terminus was purified as described in (3).

Recombinant *Homo Sapiens* α1A/βIII tubulin was expressed in SF9 cells using baculovirus and purified as described previously (4, 5). E254A and E254D mutations were introduced in α-tubulin by QuickChange mutagenesis and mutated tubulin was purified the same as wild-type tubulin.

TIRF microscopy assays of GDP or GTP-tubulin incorporation into spastin nano-damaged non-stabilized GDP-microtubules.

Porcine brain tubulin (Cytoskeleton Inc.) was used in all assays unless stated otherwise. For these experiments, tubulin was cycled, the polymer pellet was washed several times with buffer containing no nucleotide and resuspended in BRB80 with no nucleotide followed by one additional step of buffer

exchange using a concentrator. Fresh 1 mM GDP or GTP were added to the buffer when perfusion was prepared. All GMPCPP seeds from which microtubules were grown were assembled from human unmodified tubulin affinity purified (6) from tsa201 cells (7, 8) because unmodified microtubules are poor substrates for spastin (9). Dynamic microtubule extensions were grown at 16 μM tubulin with 10% Hilyte 647-labelled tubulin for 12 minutes at 30°C. Dynamic microtubules were capped with 3 μM tubulin with 15% Hilyte 647-labeled tubulin supplemented with 1 mM GMPCPP for 1.5 minutes and washed out with severing assay buffer (1X BRB80 supplemented with 50 mM KCl, 1 mM ATP, 0.1% methylcellulose 4000 cP, and oxygen scavengers)(10, 11). A pre-perfusion of 12 μM GDP-tubulin (containing 15% Hilyte488-tubulin) supplemented with 1 mM GDP or 12 μM tubulin GDP-tubulin (containing 15% Hilyte 488-tubulin) supplemented with 1 mM GTP in BRB80 was perfused into the chamber. Then, 12 μM GDP-tubulin (containing 15% Hilyte 488-tubulin) supplemented with 1 mM GDP or 12 μM GDP-tubulin (containing 15% Hilyte488-tubulin) supplemented with 1 mM GTP in severing assay buffer with 10 nM spastin and 1 mM ATP was perfused as images were acquired in the 488 and 647 channels simultaneously at 2 Hz (10). The experiments with 7 μM tubulin were performed the same way. Tubulin incorporation was determined by measuring the mean intensity along the microtubule lattice. The local background was subtracted from the mean intensities to obtain the background subtracted mean intensities.

Images were acquired on a Nikon Ti-E with a motorized TIRF arm. A 100x 1.49 NA objective was used. The excitation and emission were split using a 405/488/561/640 quad dichroic from Semrock. The excitation was further split using a long pass filer (640R) from Semrock and directed to one of two Andor-897 EMCCD cameras (green 525/50) or red (650LP) which were setup to acquire image simultaneously when triggered by an AOTF that gated the excitation. An additional 2x magnification is introduced by the emission splitting optics and a final pixel size of 0.077 μm was used. The imaging chamber was temperature controlled using an objective heater (Bioptechs) to maintain 30+/-0.5°C. The system was run using the open-source Micromanager software (12). A standard microscope slide with a grid of diffraction-limited

holes was simultaneously imaged in both channels and the ImageJ plugin "gridAligner" was used to correct for the shift between channels.

Binding of EB1 to nanodamaged sites introduced by spastin in the presence of GDP- and GTP-tubulin

Dynamic microtubule extensions were grown at 16 μM tubulin with 10% Hilyte647-labelled tubulin for 12 minutes at 30°C. Dynamic microtubules were capped with 3 μM tubulin with 15% Hilyte647-labeled tubulin supplemented with 1 mM GMPCPP for 90 s and washed out with severing assay buffer (1XBRB80 supplemented with 50 mM KCl, 1 mM ATP, 0.1% methylcellulose 4000 cP, and oxygen scavengers). 12 μM GDP-tubulin or 12 μM GTP-tubulin with 50 nM EB1-GFP in severing assay buffer was perfused into the chamber followed by introduction of 5 nM spastin in the presence 12 μM GDP-tubulin or 12 μM GTP-tubulin and 50 nM EB1-GFP in severing assay buffer. Images were acquired in the 488 and 647 channels simultaneously at 2 Hz. The lattice intensity was measured using imageJ using a 5-pixel wide line and excluding the bright EB1 comets at the tips. The line ROI was then moved to a region next to the lattice to calculate the background. The mean background was then subtracted from the mean lattice intensity to yield the mean background corrected lattice intensity. This procedure was done at frame 1 (before perfusion) and again at frame 43 (21.5 seconds) after the perfusion ended.

Spastin binding to GDP and GMPCPP microtubules

For Figure S3, double-cycled, GMPCPP-stabilized, unmodified microtubules with 2% biotinylated tubulin were used as seeds. The GDP segment was polymerized by addition of 23 μM brain tubulin and 0.1 mM GTP to the seeds and incubation for 20 min at 37°C. The GMPCPP segment was polymerized by addition of 2 μM brain tubulin and 0.5 mM GMPCPP to seeds with GDP extensions and incubated for 1 hr. To distinguish between GDP and GMPCPP segments, the former was labeled with 20% HiLyte647-tubulin and the latter with 5% HiLyte647-tubulin. The opposite combination of labeling ratios was also tested to make sure that the type of fluorophore does not affect spastin binding. Microtubules were immobilized in

the chamber and imaged at 30°C in assay buffer containing 47 mM PIPES pH 6.8, 3.3 mM HEPES pH 7.0, 50 mM KCl, 2.2 mM $MgCl_2$, 1.3 mg/ml casein, 0.6 mM EGTA, 2.5% glycerol, 9.1 mM BME, 0.2 mM TCEP, 1 mM ATPγS, 1% Pluronic F127, 0.1% methylcellulose 4000 cP, 20 mM glucose, glucose oxidase and catalase. Then, 4 nM Atto488-labeled spastin was perfused into the chamber in assay buffer at 30°C during image acquisition. Images were collected continuously in the 488 nm and 640 nm channels at 100 ms exposure. Spastin binding was quantified as background-subtracted average intensity along microtubule in the 488 nm channel on a sum of frames 250 to 600.

Spastin expression plasmids for cellular studies

Full-length human M87 spastin with a C-terminal FLAG tag was expressed from a CMV promoter as previously described (13). Full-length human M87 spastin with a C-terminal-FLAG-myc-mCherry tag was cloned into the Dox-inducible Xlone piggybac plasmid [gift from Xiaojun Lian (Addgene plasmid # 96930; http://n2t.net/addgene:96930 ; RRID:Addgene_96930)]. The K388R mutant was cloned from the WT construct by site-directed mutagenesis, single-fragment Gibson assembly.

Endogenous EB1 localization in cells overexpressing spastin

PtK2 cells (ATCC) were cultured at 37°C with 5% CO_2 in Opti-MEM Reduced Serum Medium with GlutaMAX (Fisher Scientific), supplemented with 10% fetal bovine serum, 25 mM HEPES, and Penicillin-Streptomycin. Cells were plated onto plasma-cleaned 22 x 22 mm #1.5 coverslips in antibiotic-free media, and 24 hours later, cells were transfected with 3 µg of plasmid DNA and 9 µL of Fugene HD (Promega), according to the manufacturer's instructions. Eighteen hours after transfections, the media was removed and a -80°C solution of 90% Methanol, 3.2% Formaldehyde, 50 µM $NaHCO_3$ pH 9.0 was added to the coverslips; cells were fixed for 10 minutes at -20°C. Cells were then rehydrated in PHEM-T (60 mM PIPES, 25 mM HEPES, 10 mM EGTA, 2 mM $MgCl_2$, 0.5% Triton X-100) for 5 minutes and then washed three times for 5 minutes in PHEM-Wash (60 mM PIPES, 25 mM HEPES, 10 mM EGTA, 2 mM $MgCl_2$, 0.1%

Triton X-100). Cells were blocked in AbDil (1x TBS, 0.1% Triton X-100, 2% BSA, 0.1% sodium azide) for one hour. Primary antibodies were diluted in AbDil at the following dilutions: rat anti-EB1 (Abcam ab53358; 1:200), mouse anti-α-tubulin (Sigma-Aldrich DM1A; 1:1000), and Rabbit anti-DYKDDDDK (Genscript; 1:200), and coverslips were inverted on a drop of the primary antibody solution and incubated in a humidified chamber for one hour at room temperature. Coverslips were washed four times for five minutes in PHEM-Wash and then incubated for one hour on a drop of secondary antibody solution containing highly cross-subtracted donkey anti-rat-AlexaFluor 488 (Jackson Immunoresearch; 1:300), cross-subtracted goat anti-Rabbit-AlexaFluor 555 (Fisher Scientific; 1:300), and highly cross-subtracted donkey anti-mouse-AlexaFluor 647 (Jackson Immunoresearch; 1:300). Images were acquired on a Nikon Eclipse Ti microscope outfitted with a 60x 1.49 NA objective, Hamamatsu Flash 4.0 camera, an Agilent four laser combiner (405, 488, 561, 640 nm), and Yokogawa CSU-X1 spinning disk scan head. Acquisition times for each image were as follows: 488 nm (EB1 excitation), 400 ms, 561 nm (spastin-FLAG excitation), 200 ms, 640 nm (tubulin excitation), 200 ms. To quantitate EB1 microtubule binding, 5-pixel wide lines were drawn along single microtubules from their plus-ends, excluding the comets. The measurements were made on single microtubules, and the ends (EB comets) were excluded. In cases where a comet from an adjacent microtubule overlapped, that region was excluded from the analysis. Comets are distinguished from the EB1 islands by their bright signal (several fold higher than the islands) and that they occur only at the tips of microtubules. EB1 intensities were measured along these lines and corrected using the local background. Microtubule bundles were excluded from quantification.

EB1-mNeonGreen CRISPR knock-in and spastin-mCherry inducible expression cell line for live-cell imaging.

For knock-in of EB1, a gRNA target (CTGGACCAGCAGAGCAACAT CGG) slightly downstream of the stop codon of human EB1 gene was selected and cloned into a U6-driven gRNA vector as reported (14). For homology repair template, about 600-800bp of both upstream and downstream of the stop codon of

EB1 gene were cloned from U2OS genomic DNA. To facilitate selection, a puromycin resistant coding sequence was added making a final homologous repair backbone as following: 5'homology arm – linker – mNeonGreen - bGH poly(A) signal - EF-1 alpha promoter – Puromycin - synthetic poly(A) signal/transcriptional pause - 3'homology arm. The sequence of the linker between EB1 and mNeonGreen is FEGGGSGGGS. To get knock-in cells, U2OS cells were transfected with wild-type Cas9, gRNA, homology repair plasmids using Lonza Cell Line Nucleofector Kit V. 5 days post-transfection, puromycin (1 ug/mL final) was added into the medium and cells were further cultured for 5 days before being sorted into single cells. Western blotting using both anti-EB1 antibody (Proteintech, 17717-1-AP, 1:500 for WB) and anti-mNeonGreen (Chromotek, 32F6, 1:500 for WB) antibodies were used for final validation of positive knockin after expansion of the single-cell clones (Figure S2A). An EB1-mNG heterozygous clone was chosen for all downstream studies. To generate a stable-cell line population additionally harboring dox-inducible spastin, cells were then transfected with the M87spast-mCherry Xlone plasmid alongside a PiggyBac transposase vector (Hera Biolabs, Inc.) for genome integration of the transgene using Fugene HD (Promega) and blasticidin-selected beginning the following day. To generate cell lines expressing mutated spastin, mutations were introduced into the M87spast-mCherry Xlone plasmid by QuickChange mutagenesis and the following steps were the same as for the wild-type spastin. Cells carrying wild-type spastin were then FACs-sorted in presence of doxycycline (2 μg/ml) to further enrich for inducible spastin expressors.

Endogenous and mCherry-tagged spastin levels at 0 or 2 μg/ml doxycycline were assessed by Western blot (Figure S2B, C). For preparation of whole-cell lysate, cells were washed with PBS and lysed in RIPA buffer (50 mM Tris-HCl, 150 mM NaCl, 0.1% SDS, 0.5% NaDoc, 1% TritonX-100 pH 7.5) with addition of cOmplete™ Mini EDTA-free Protease Inhibitor Cocktail Tablets (Sigma), incubated on ice for 15 minutes with intermittent vortexing and clarified at 14000 x g for 20 min at 4°C. Lysates were resolved by SDS PAGE and transferred to a nitrocellulose membrane. The membrane was blocked in 5% milk in PBST for

1 hour and then incubated with primary antibodies: anti-spastin (Abcam, ab77144), and anti-GAPDH to normalize spastin signal (Cell Signaling Technologies; mouse-anti-GAPDH D4C6R-97166 and rabbit-anti-GAPDH 14C10-#2118) overnight at 4°, followed by 3 washes with PBST and 1 h incubation at room temperature with secondary antibodies (Licor IRDyes 680 and 800CW goat-anti-mouse and goat anti-rabbit igG (H+L) LIC-926-68020, LIC-926-32211). The membrane was washed 3 times with PBST followed by a final wash with PBS and imaged using the Odyssey CLx system.

Live-cell imaging of EB1-mNeonGreen CRISPR knock-in cells overexpressing spastin

48 h prior to imaging, EB1-mNG/spastin-mCh cells cultured at 37°C with 5% CO_2 were seeded onto fibronectin-coated (Sigma) 8-well glass bottom chambers (Ibidi 80827) in DMEM (1x) + GlutaMAX-I (Gibco 10564029) and 10% Tet-system approved fetal bovine serum (Gibco A4736201). 24 h prior to imaging, doxycycline-containing media (2 μg/ml) (Clontech) was added to the cells. On the day of imaging, cells were washed one time with FluoroBrite DMEM (Gibco A1896701) and freshly-prepared imaging media (FluoroBrite DMEM with 10% FBS, 1X Pen/Strep, 1X L-glutamine, 0.1% DL-lactate (Sigma) and ProLong Live Antifade Reagent per manufacturer's instructions (ThermoFisher P36975) supplemented with fluorogenic microtubule probe SiR-Tubulin (1:2000, 500 nM) (Cytoskeleton, Inc), verapamil (1:2000, 10 μM) (Cytoskeleton, Inc) and doxycycline (2 μg/ml) was added back and incubated for approximately 1 h prior to imaging. To measure microtubule growth rates, cells were imaged in the same conditions but omitting SiR-Tubulin and verapamil.

Images were acquired on a Nikon Ti2 microscope outfitted with Yokogawa SoRa CSU-W1 spinning disk unit with 2.8x SoRa magnifier, 100x oil immersion objective lens (N.A. 1.45) and Hamamatsu ORCA-Fusion BT sCMOS camera. Images were acquired with 488 nm (EB1-mNG excitation), 561 nm (spastin-mCh excitation) and 640 nm (SiR-tubulin excitation) diode lasers with 400 ms exposure in each channel at 1.66 s interval. As not all cells in the population turn on spastin in response to doxycycline treatment, cells

were binned into spastin and non-spastin expressing cells based on spastin-mCherry signal detection (quantified as mean fluorescent intensity of a cytoplasmic ROI relative to background signal). Average mCherry signal of spastin-expressing cells used for quantification of EB1 microtubule binding was not significantly different between wild-type and mutant cell lines. To quantify the extent of EB1 microtubule binding, 10-pixel wide lines were traced along microtubules, excluding EB1-mNG comets, microtubule bundles and crossovers, and the intensity of EB1 was averaged along these lines and corrected using a local background subtraction to obtain per cell averages of EB1 lattice binding. To quantify microtubule growth rates, mNeonGreen-EB1 comets were tracked using UTrack 2.2.0 software (15) and only tracks with at least 2 μm displacement were included for further analysis to filter out incorrectly detected comets. Images were binned 2x2 to a 46 nm pixel size before analysis because UTrack does not track correctly comets in images with the smaller pixel size of 23 nm.

Nucleotide content analysis

To analyze the nucleotide content in microtubules, brain tubulin, wild-type recombinant tubulin α1A/βIII and GTP hydrolysis deficient tubulin mutants (E254A and E254D) were polymerized from GMPCPP microtubule seeds and subjected to nucleotide extraction and HPLC analysis based on a modified published protocol (16). GMPCPP-stabilized microtubule seeds were prepared as described (17), flash-frozen in small aliquots and stored at -80°C. Before use, the seeds were diluted to 1.5 mg/ml in warm BRB80 buffer (80 mM PIPES, 1 mM EGTA, 1 mM $MgCl_2$, pH 6.8) and placed at 37°C for one hour. 150 μg of each tubulin variant was used for the polymerization reaction at final tubulin concentration of 15 μM. Reactions were performed in BRB80 buffer supplemented with 11% DMSO, 1 mM GTP and 1 mM $MgCl_2$. Tubulin with supplements was pre-incubated on ice for 5 min, then transferred to 37°C for 1 min. 0.15 μM GMPCPP-stabilized seeds were added, and the polymerization was carried out for one hour. The samples were centrifuged at 278,000 x g for 10 min at 37°C. Microtubule pellets were washed three times with warm BRB80 buffer and resuspended in 10 mM Tris-HCl pH 7.5 to a final concentration 20 μM. 60 μls were

used for nucleotide extraction by addition of 0.8% (vol/vol) $HClO_4$. The pH was adjusted to ~ 5.5 with 2M sodium acetate (final concentration 400 mM). Precipitated protein was removed by centrifugation at 15,000 x g at 4°C for 10 min. Collected supernatants (60 µl, containing nucleotides extracted from 100 µg of microtubules) were subjected to HPLC analysis on a Supelcosil LC-18-T column (4.6x150 mm, 3 µm pore size) in isocratic elution at 0.7 ml/min in 100 mM K_2HPO_4/KH_2PO_4 (pH 6.5), 10 mM tetrabutylammonium bromide, 7% (vol/vol) acetonitrile and detection at 260 nm. GDP and GTP standards were HPLC analyzed in the same conditions. Chromatograms were plotted using Prism software.

Microtubule repair assays with recombinant tubulin

Double-cycled, GMPCPP-stabilized brain microtubules containing 1% biotinylated tubulin and 15% HiLyte647-tubulin (Cytoskeleton) were polymerized as in (11, 17). We note that the fluorescent signal from the HiLyte647-tubulin is not uniform along the lattice and cannot be used to quantify or detect nanodamage sites which are diffraction limited. Microtubules were immobilized in the chamber made of a silanized slide and coverslip using NeutrAvidin (Thermo Fisher Scientific) (1, 5) and imaged at RT using TIRF (10) in severing buffer containing 47 mM PIPES pH 6.8, 3.3 mM HEPES pH 7.0, 50 mM KCl, 2.2 mM $MgCl_2$, 1.3 mg/ml casein, 0.6 mM EGTA, 2.5% glycerol, 9.1 mM 2-mercaptoethanol, 0.8 mM DTT, 1 mM ATP, 1% Pluronic F127, 20 mM glucose, glucose oxidase and catalase. Microtubules were then incubated with 20 nM spastin for 30 s in severing buffer. This was followed by perfusion of recombinant tubulin together with 100 nM EB1-GFP in repair buffer consisting of 52 mM PIPES pH 6.8, 10 mM HEPES pH 7.5, 50 mM KCl, 0.65 mM $MgCl_2$, 0.65 mM EGTA, 0.5 mM GTP, 1 mM ADP, 1% Pluronic F127, 2.7 mg/ml casein, 2.1 mM 2-mercaptoethanol, 0.1 mM DTT, 0.75% glycerol, 20 mM glucose, glucose oxidase and catalase. Tubulin concentrations were 5 µM for wild-type, 2.5 µM for E254D and 1.2 µM for E254A α1AβIII-tubulin. These concentrations were used so that the microtubules are growth-matched i.e. they have the same rates of tubulin addition at microtubule ends (16). Movies were acquired in TIRF with continuous 100 ms exposure with the 488 laser and imaged with an EMCCD camera (Andor). Positions of microtubules

were determined from a snapshot in the 640 nm channel as above. Both 488 and 640 lasers were set to 20 mW. To measure the fluorescent signal of single EB1-GFP molecules, diluted EB1 was immobilized directly on the surface of silanized glass, unbound molecules were washed out with BRB80 (80 mM PIPES pH 6.8, 1 mM $MgCl_2$, 1 mM EGTA) and movies were collected in repair buffer using the same settings as those used for repair assays.

EB1-GFP islands analysis with recombinant tubulin repair

EB1-GFP islands were manually tracked on kymographs made with a 3-px-wide line selection along the microtubules in the 488 nm channel. Residual diffuse EB1 binding along the microtubule was the same for all the conditions and it is not due to the bleed through from the microtubule channel because microtubules were invisible in the 488 nm channel in the absence of EB1-GFP. EB1 binding events occurred very rarely in the non-damaged control, while, in contrast, they were frequent on the enzyme nanodamaged microtubules, and at least two times brighter than the residual lattice binding. The lifetimes of EB1-GFP islands did not fit to a single exponential model and we do not have enough insight to fit it to a different model, therefore we report median lifetime. The lifetimes were not corrected for photobleaching since there was an excess of EB1-GFP and one EB1-positive island was bound by multiple EB1-GFP molecules. To quantify the maximum number of EB1 molecules at any time in an EB1 island, the background-subtracted highest intensity of each EB1 island was divided by the average background-subtracted intensity of a single EB1-GFP molecule immobilized on glass imaged under the same conditions.

TIRFM-based microtubule dynamics assays

Dynamic microtubule extensions were grown at 12 µM tubulin with 10% Hilyte 647-labelled tubulin for 10 minutes at 30°C. Then, 7 µM or 12 µM tubulin of which 10% is Hilyte 647-labelled, 50 nM EB1-GFP and 0 or 20 nM spastin with 1 mM ATP or 1 mM ATPγS in 1XBRB80 supplemented with 50 mM KCl, 1 mM GTP, 0.1% methylcellulose 4000 cP, and oxygen scavengers was perfused into the chamber as images

were acquired in the 488 and 647 channels simultaneously at 2 Hz. Microtubule dynamics were quantified for up to 3 min after spastin perfusion. After 3 minutes many microtubules became bundled (due to new microtubules generated by severing), and spastin activity started to decrease. In the spastin and ATP 12 μM tubulin condition, the chambers became too crowded to analyze after ~ 1 minute. Rescue frequencies were quantified as the number of rescues per depolymerization time. Depolymerization lengths were quantified as the total length a microtubule depolymerized before rescuing. Only events that depolymerized for at least 5 frames were used in the depolymerization length measurement to account for error in measuring short lengths of very rapidly rescuing events. Kymograph analysis was difficult to perform due to microtubule severing and microtubule fluctuations and thus each microtubule was followed manually, and polarity was assigned by tracking it from the initial severing event when the microtubule was still connected to its seed. Most rescue events were also marked by the immediate appearance of a bright EB1 comet (brighter than the EB1 at repair sites); if they were not, kymographs for a few frames (during which the microtubule did not fluctuate significantly) were used to determine if the microtubule was growing again. In almost all the latter cases the microtubules gained a bright EB comet a few frames later, and the rescue was scored at the time depolymerization stopped. The mean and standard deviation of the EB1 lattice signal in 7x7 pixel regions on the lattice in the no enzyme condition was measured and used to determine the threshold for EB1 puncta in the + enzyme condition is. An EB1 punctum was counted if it had a mean intensity (in a 7x7 pixel box) at least 2.5 standard deviations above the mean lattice intensity in the no damage condition. Once a punctum was detected, its mean intensity in the following frame was measured until it dropped below the detection criteria. The number of frames it was tracked for was then reported as the puncta lifetime. Microtubule end stability was scored manually by looking at the ends after a microtubule was severed. If it did not depolymerize and it acquired a growing EB1 comet within 2 frames it was scored as stable. Assays with different tubulin concentration (Figure S5) were performed by first growing microtubules at 12 μM tubulin consisting of 15% HiLyte-647 tubulin, and then perfusing in either 3, 5, or 12 μM 15% HiLyte-488 tubulin with 20 nM spastin and 1 mM ATP.

IRM-based microtubule dynamics assays

For the IRM assays, dynamic microtubule extensions were grown at 12 μM tubulin for 12 minutes at 30°C in 1XBRB80 supplemented with 50 mM KCl, 1 mM GTP, 0.1% methylcellulose 4000 cP, 0.5% Pluronic F127, 0.1 mg/ml casein and oxygen scavengers with 1 mM ATP or 1 mM ATPγS. Then, 50 nM EB1-GFP, 7 μM or 12 μM tubulin, and 0, 20 or 40 nM spastin was perfused into the chamber in the same buffer as the first perfusion. The IRM setup was configured as described in reference (18). Images were acquired in the 488 and IRM channels simultaneously at 10 Hz. For simultaneous 488 and IRM imaging, a Di02-R405/488/561/635 dichroic was placed in the microscope turret. Excitation was provided simultaneously with a 488 nm laser (Coherent sapphire) and a M617L3 LED from ThorLabs. Emission light was split using a Di02-R594 (Semrock). A 514/30 filter was used on the GFP channel and a FF01-593LP was used on the IRM channel (5). To assign polarity, microtubules were imaged in the growth phase before introducing spastin. The IRM image was background-subtracted. Background was determined from the median of multiple fields of view after the assays, from each chamber separately. The 488 and IRM channels were aligned using the ImageJ plugin "gridAligner". Dynamic parameters were scored as described above for the TIRF-based microtubule assays. For examples see Figure S7.

Statistical analysis

Each condition was tested in independent chambers on different days. Data were plotted and statistical analysis performed in Prism (Graphpad Software). 2-tailed t-test and Mann-Whitney test were used for normally- and non-normally distributed data, respectively. Ordinary one-way ANOVA or Kruskal-Wallis tests were used to correct for multiple comparisons for normally- and non-normally distributed data, respectively. The statistical test used for each dataset is specified in the figure legends. After the data were analyzed, an *a priori* statistical testing using the two group means, sizes, and standard deviations was used

to determine whether enough sampling was performed for the tests to achieve adequate power. In all cases the size of our data sets yielded statistical test power of at least 90%.

SI References

1. N. E. Ziolkowska, A. Roll-Mecak, In vitro microtubule severing assays. *Methods Mol Biol* **1046**, 323-334 (2013).
2. J. M. Antos *et al.*, Site-specific protein labeling via sortase-mediated transpeptidation. *Current protocols in protein science* **89**, 15.13. 11-15.13. 19 (2017).
3. A. J. Albee, C. Wiese, Xenopus TACC3/maskin is not required for microtubule stability but is required for anchoring microtubules at the centrosome. *Mol Biol Cell* **19**, 3347-3356 (2008).
4. A. Vemu *et al.*, Structure and Dynamics of Single-isoform Recombinant Neuronal Human Tubulin. *J Biol Chem* **291**, 12907-12915 (2016).
5. J. Chen *et al.*, α-tubulin tail modifications regulate microtubule stability through selective effector recruitment, not changes in intrinsic polymer dynamics. *Dev Cell* **56**, 2016-2028. e2014 (2021).
6. P. O. Widlund *et al.*, One-step purification of assembly-competent tubulin from diverse eukaryotic sources. *Mol Biol Cell* **23**, 4393-4401 (2012).
7. A. Vemu, C. P. Garnham, D.-Y. Lee, A. Roll-Mecak, Generation of differentially modified microtubules using in vitro enzymatic approaches. *Methods in enzymology* **540**, 149-166 (2014).
8. A. Vemu, J. Atherton, J. O. Spector, C. A. Moores, A. Roll-Mecak, Tubulin isoform composition tunes microtubule dynamics. *Molecular biology of the cell* **28**, 3564-3572 (2017).
9. M. L. Valenstein, A. Roll-Mecak, Graded control of microtubule severing by tubulin glutamylation. *Cell* **164**, 911-921 (2016).
10. A. Vemu *et al.*, Severing enzymes amplify microtubule arrays through lattice GTP-tubulin incorporation. *Science* **361** (2018).
11. A. Vemu, E. Szczesna, A. Roll-Mecak, In Vitro Reconstitution Assays of Microtubule Amplification and Lattice Repair by the Microtubule-Severing Enzymes Katanin and Spastin. *Methods Mol Biol* **2101**, 27-38 (2020).
12. A. D. Edelstein *et al.*, Advanced methods of microscope control using μManager software. *Journal of biological methods* **1** (2014).
13. S. H. Park, P. P. Zhu, R. L. Parker, C. Blackstone, Hereditary spastic paraplegia proteins REEP1, spastin, and atlastin-1 coordinate microtubule interactions with the tubular ER network. *J Clin Invest* **120**, 1097-1110 (2010).
14. P. Zheng *et al.*, DNA damage triggers tubular endoplasmic reticulum extension to promote apoptosis by facilitating ER-mitochondria signaling. *Cell research* **28**, 833-854 (2018).
15. K. Jaqaman *et al.*, Robust single-particle tracking in live-cell time-lapse sequences. *Nature methods* **5**, 695-702 (2008).
16. J. Roostalu *et al.*, The speed of GTP hydrolysis determines GTP cap size and controls microtubule stability. *Elife* **9** (2020).
17. C. Gell *et al.*, Microtubule dynamics reconstituted in vitro and imaged by single-molecule fluorescence microscopy. *Methods in cell biology* **95**, 221-245 (2010).

18. Spector, J.O., Chen, J., Szczesna, E. and Roll-Mecak, A., 2025. Multicamera simultaneous total internal reflection and interference reflection microscopy. *Journal of Microscopy*, *298*(1), pp.10-16.
